# Effectiveness of an impairment-based individualized rehabilitation program using an iPad-based software platform

**DOI:** 10.3389/fnhum.2014.01015

**Published:** 2015-01-05

**Authors:** Carrie A. Des Roches, Isabel Balachandran, Elsa M. Ascenso, Yorghos Tripodis, Swathi Kiran

**Affiliations:** ^1^Aphasia Research Laboratory, Speech, Language, and Hearing Sciences, Sargent College, Boston UniversityBoston, MA, USA; ^2^Department of Biostatistics, School of Public Health, Boston UniversityBoston, MA, USA

**Keywords:** aphasia, stroke, traumatic brain injury, iPad-based rehabilitation, Constant Therapy, impairment-based, individualized rehabilitation

## Abstract

The delivery of tablet-based rehabilitation for individuals with post-stroke aphasia is relatively new, therefore, this study examined the effectiveness of an iPad-based therapy to demonstrate improvement in specific therapy tasks and how the tasks affect overall language and cognitive skills. Fifty-one individuals with aphasia due to a stroke or traumatic brain injury (*TBI*) were recruited to use an iPad-based software platform, Constant Therapy, for a 10 week therapy program. Participants were split into an experimental (*N* = 42) and control (*N* = 9) group. Both experimental and control participants received a 1 h clinic session with a clinician once a week, the experimental participants additionally practiced the therapy at home. Participants did not differ in the duration of the therapy and both groups of participants showed improvement over time in the tasks used for the therapy. However, experimental participants used the application more often and showed greater changes in accuracy and latency on the tasks than the control participants; experimental participants' severity level at baseline as measured by standardized tests of language and cognitive skills were a factor in improvement on the tasks. Subgroups of task co-improvement appear to occur between different language tasks, between different cognitive tasks, and across both domains. Finally, experimental participants showed more significant and positive changes due to therapy in their standardized tests than control participants. These results provide preliminary evidence for the usefulness of a tablet-based platform to deliver tailored language and cognitive therapy to individuals with aphasia.

## Introduction

About 795,000 Americans suffer a new or recurrent stroke each year, which is the leading cause of serious long term disability in the US. Additionally, there are over 1.7 million new cases of brain injury each year. Aphasia (or the impairment of language) is the effect of a brain injury, most often from a stroke in the left (and sometimes right) hemisphere of the brain. It is estimated that approximately 200,000 individuals acquire aphasia each year (www.aphasia.org). Typically, even though there is improvement in language function in the first months after stroke, most individuals continue to present with aphasia (Pedersen et al., 2004) and likely require long-term aphasia rehabilitation. There is a large body of research suggesting that individuals with aphasia continue to improve their communication abilities with rehabilitation (Raymer et al., 2008; Allen et al., 2012; Teasell et al., 2012). However, for most patients, insurance only covers the acute in-hospital and initial rehabilitation services. Consequently, it is a huge practical problem to provide the continued communication rehabilitation that these individuals require.

One way to achieve continued rehabilitation is through the use of technology. Computerized rehabilitation is becoming more frequently utilized and has been shown to facilitate improvements in individuals who have had a brain injury or mild cognitive impairments. While several rehabilitation tools are available on the internet, only a few are discussed here. One such program is CogMed (Pearson Company, Scandinavia, Sweden), which is software implemented to improve working memory abilities in individuals with brain injury. Westerberg, Jacobaeus et al. studied the effectiveness of CogMed software in 18 stroke participants split into a control group and a treatment group. After 5 weeks, treatment participants showed more improvement on untrained measures of working memory and attention than control participants. Participants also self-reported fewer cognitive problems after the treatment (Westerberg et al., 2007). Lundqvist, Grundström et al. used CogMed software with 21 participants who had a traumatic brain injury (*TBI*) or a stroke, split into control and treatment groups. After 5 weeks, participants showed improvement in the trained tasks and self-reported fewer cognitive failures, greater occupational performance, and increased satisfaction (Lundqvist et al., 2010). Johansson and Tornmalm studied the effects of CogMed on 18 participants with working memory deficits. Results showed that after training, participants improved on the trained task, especially when the deficit was more impaired at baseline. Participants also self-reported fewer cognitive problems in daily life. All results were maintained at a 6 month follow up (Johansson and Tornmalm, 2012). Finally, Åkerlund et al. studied the effect of the software on 47 *TBI* participants, split into an intervention group and a control group. Both groups received integrated rehabilitation but the intervention group received additional CogMed therapy. Both groups showed improvement in working memory and participants reported fewer depressive symptoms (Akerlund et al., 2013). In general, it appears that training working memory through a software training program improves working memory functions and some other self-reported gains in daily functioning.

Another online-based tool for rehabilitation is Lumosity (Lumos Lab, San Francisco, CA), also available on the internet, which targets attention, processing speed, visual memory in older individuals with mild cognitive impairments. Finn and McDonald studied 16 participants with mild cognitive impairments who completed 30 sessions of training with Lumosity. Participants improved on the trained tasks and showed some evidence of generalization to a measure of visual sustained attention (Finn and McDonald, 2011). Zickefoose et al. studied four *TBI* participants using both Lumosity and Attention Processing Training-3 (Lash & Associates Publishing/Training, Inc., Youngsville, NC). Both interventions resulted in improvements on the trained tasks in all participants and limited generalization to standardized measures was demonstrated in one of the participants (Zickefoose et al., 2013).

Finally, Posit Science (Posit Science, San Francisco, CA) is targeted to improve auditory processing speed in individuals with mild cognitive impairment. Barnes et al. looked at the effectiveness of this tool in 47 participants with mild cognitive impairment. The results, though not statistically significant, showed that the experimental group scored higher than the control group (which only completed passive computer activities) in verbal learning and memory measures (Barnes et al., 2009). In general, these programs have been found to result in gains on the trained tasks with limited generalization to tasks that engage similar processing skills.

Computer rehabilitation has also been shown to facilitate improvements in naming with chronic aphasia patients. Doesborgh et al. tested cued naming on 18 individuals with naming deficits; eight participants received 10–11 h of Multicue, a computer program for word finding therapy, and 10 participants received no therapy. Results showed that participants who received Multicue therapy improved on the Boston Naming Test, but the changes were not significant when compared to the control group (Doesborgh et al., 2004). Fink et al. used another computerized cued naming therapy called MossTalk Words (Moss Rehabilitation Research Institute, Elkins Park, Pennsylvania) in their study of six participants with aphasia. Three participants received full clinician guidance while using the computer program and three participants used the computer program with partial independence. Both groups of participants showed positive effects of the training (Fink et al., 2002). Palmer et al. studied 28 individuals with aphasia who completed 5 months of treatment; 15 participants used a self-managed computer word finding treatment and 13 completed usual care treatment (everyday language activities). The researchers found the computer treatment group showed significantly more improvement on their naming ability from the baseline test than the usual care group (Palmer et al., 2012). Pedersen and Vinter studied three individuals with anomia using an unsupervised computerized rehabilitation and found that all participants showed varying degrees of improvement (Pedersen et al., 2001). Ramsberger and Marie studied another computer-based cued naming therapy that was self-administered with some guidance from a clinician. Four individuals with aphasia participated; two received therapy twice a week and two received therapy five times a week. The researchers found that, regardless of the intensity of the therapy, three of the four participants showed evidence of improved naming (Ramsberger and Marie, 2007).

All of these studies have found the beneficial effects of a computer based naming therapy; however, they have only examined a limited population size. In contrast, Katz and Wertz studied 55 chronic aphasic individuals who were randomly assigned to one of three groups: computerized reading treatment that involved visual matching and reading comprehension tasks, computer stimulation that involved non-verbal games and cognitive rehabilitation tasks, or no stimulation. The researchers found that the computerized reading treatment participants showed more significant improvement than the other two groups on the Overall and Verbal subtests of the Porch Index of Communicative Ability as well as on the Aphasia Quotient and the Repetition subsections of the Western Aphasia Battery (Katz and Wertz, 1997).

To summarize, there are two groups of studies that have been reviewed above, one group of studies have examined computer rehabilitation with reasonably sized groups of patients with cognitive impairments, but the patients received standardized cognitive-based interventions. The second group of studies, particularly in the aphasia literature, have examined fewer participants but appeared to have tailored the intervention for the particular participants. Both sets of studies provide promising preliminary results for the use of technology in the rehabilitation of patients with brain injury.

More recently, the advent of tablet based devices, such as the iPad, has proved to be promising for rehabilitation (Holland, 2014; Hoover and Carney, 2014; Kiran et al., 2014; Kurland, 2014; Kurland et al., 2014; Ramsberger and Messamer, 2014; Szabo and Dittelman, 2014). For instance, Kurland et al. studied the use of an iPad-based home practice program for maintaining and improving object and verb naming following a 2 week intensive language therapy program. The researchers found that all five participants who completed the home practice program maintained the advances made from the intensive therapy while also learning new words over the 6 month period of home practice. Although their results were promising, the researchers noted there was often a need for the software to increase in task difficulty when their participants showed improvement, demonstrating the need of tailored therapy for individuals (Kurland et al., 2014).

While there is increased awareness and momentum for applying computer technology in aphasia rehabilitation, there are also several cautions. First, the standards for providing evidence for technology-based rehabilitation applications should be no different than traditional rehabilitation outcome measures. On the other hand, the effectiveness of traditional rehabilitation outcomes is itself a topic of debate. While some recent meta-analyses (Allen et al., 2012; Teasell et al., 2012) argued for the beneficial effect of rehabilitation in chronic stroke survivors, another recent influential study suggested that rehabilitation was no more effective than everyday communication in acute stroke survivors (Bowen et al., 2012). Additionally, a comprehensive review of aphasia therapy completed by Kelly et al. found difficulties in drawing overall conclusions about the effectiveness of therapies due to individual patient variability, such as aphasia profile, age, and time post onset of injury (Kelly et al., 2010). Therefore, while the benefits of rehabilitation in stroke and *TBI* would seem logical, there is a need to demonstrate clear rehabilitation outcomes in large sets of stroke (and *TBI*) individuals, and our study addresses this goal. Secondly, it is not clear what the optimal dosage or intensity of therapy may be for stroke and *TBI* individuals. While some meta-analytic reviews have suggested that more therapy results in greater outcomes (Bhogal et al., 2003; Cherney et al., 2008), other studies, including a randomized controlled trial found that intensive therapy (up to 5 h per week) was no better than standard therapy (1–2 h per week) (Bakheit et al., 2007). Therefore, even though it seems logical to assume that more therapy is better, it is important to demonstrate, empirically, that it is indeed more effective. In this study, we systematically compared outcomes in the experimental group and control group that differed in the amount of therapy practice.

The goal of the current study is to determine the clinical effectiveness of using iPads to deliver personalized therapy to individuals with aphasia, to determine if a structured iPad-based therapy program that includes homework practice results in significant gains in overall communication and how individual severity profiles affect therapy outcomes. Additionally, the study examines the effectiveness of using language and cognitive therapy tasks to facilitate improvements in accuracy and latency in a group of heterogeneous post-stroke and *TBI* individuals. This study compares participants who received an individualized iPad-based therapy program practiced up to 7 days a week, 1 h of which is in the clinic, with participants who received individualized iPad-based therapy for 1 h, 1 day per week by a clinician.

The following questions were asked in this study: (1) Can an iPad-based therapy program be provided in a standardized but individualized manner. If so, what does therapy dosage and therapy compliance look like? It was hypothesized that examination of therapy time and usage for both experimental and control groups would elucidate group differences in therapy compliance. (2) Are the individualized therapy tasks for language and cognitive therapy effective for improving overall language and cognitive performance? It was hypothesized that therapy programs that are tailored to the individual's language and cognitive deficit profile (i.e., impairment based intervention) are likely to be statistically effective. Also, it was predicted that experimental participants who practice more therapy will show greater gains in therapy. (3) What are profiles of individual responsiveness to therapy? Does severity affect therapy outcomes and if so, how? It was predicted that individual responsiveness to therapy would be influenced by individual language and cognitive severity profiles. (4) What is the nature of between-task co-improvement across different therapy tasks across participants? It was hypothesized that therapy tasks that were co-assigned would co-improve, specifically the ones that were related from a linguistic/cognitive perspective. (5) Finally, what is the effect of the therapy on standardized measures and is it different between the control and experimental groups? It was predicted that all participants will show positive changes on standardized measures but for experimental participants to show more changes given the greater practice.

## Materials and methods

### Participants

**Figure 1:**
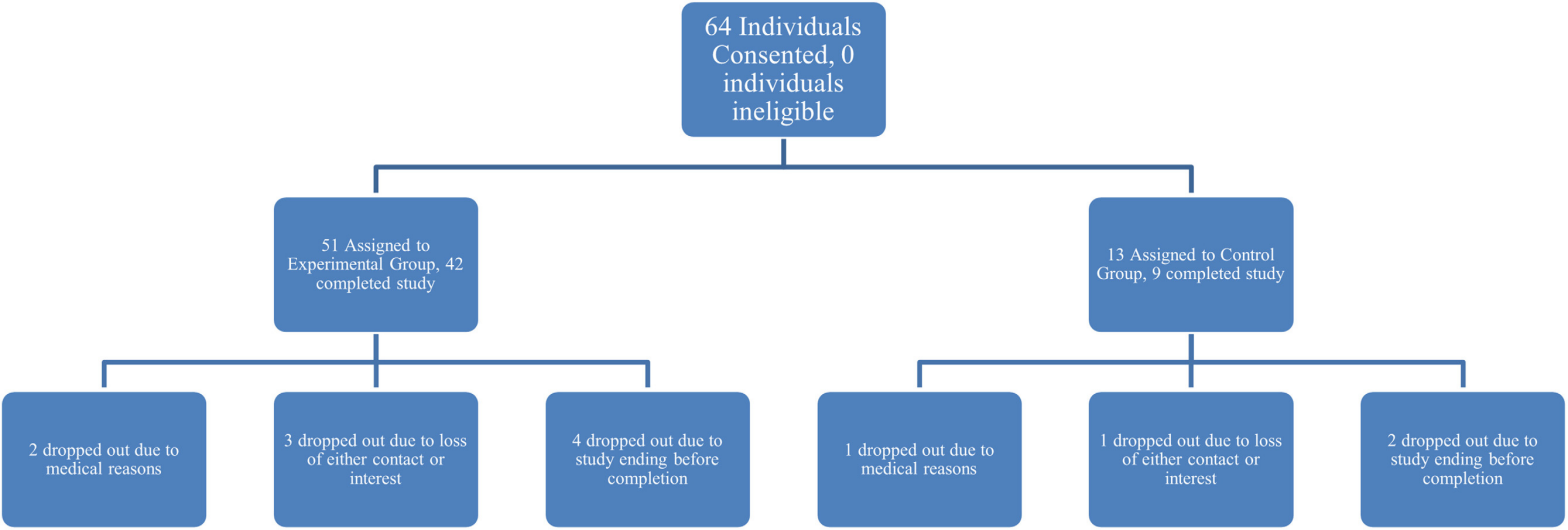
**Denotes the breakdown of participant assignment to experimental or control groups and subsequent attrition within the two groups**.

Sixty-four individuals with language and cognitive deficits were recruited from local hospitals and aphasia centers in Boston, Massachusetts and surrounding areas to participate in the study. Informed consent was obtained for all participants, in accordance with policies set forth by the Boston University Institutional Review Board. Inclusion criteria were broad, only excluding individuals with dementia or Parkinson's disease. Fifty-one individuals completed the study and 13 individuals dropped out due to personal issues, loss of contact or interest, or due to the study ending before the participants finished the therapy (Figure 1). Every fifth participant who was entered in the study was chosen to become a control participant, unless they already owned an iPad, which was the case for 22 of the 51 participants who completed the study. Of those 51 participants, nine were assigned to the control group (two female) and the remaining 42 comprised the experimental group (18 female). All participants suffered either a stroke or a *TBI*, ranging in months post onset (*MPO*) from 1 to 178 months for experimental participants (*M* = 51.4, *SD* = 47) and from 13 to 359 months for control participants (*M* = 98, *SD* = 129.7), which was significantly different (*t* = 2.76, *p* < 0.0001). This significant difference as well as the type of injury is considered in the results when applicable. Experimental participants ranged in age from 38 to 83 years (*M* = 63.6, *SD* = 10.8) and control participants ranged in age from 53 to 87 years (*M* = 67.1, *SD* = 10), which was not significantly different (*t* = 1.09, *p* = 0.87). There were five participants with a history of *TBI* and the remaining participants had suffered a stroke. Refer to Table 1 for all participants' age, *MPO*, and cause of injury.

**Table 1:** **All participants' age, *MPO*, cause of injury, and severity scores from pre-and post-therapy, separated by experimental and control group**.

**ID**	**Group**	**Age**	**MPO**	**Cause**	**R-WAB**		**CLQT**		**BNT**		**PAPT**	
					**Aphasia quotient**		**Composite severity**					
					**Pre**	**Post**	**Pre**	**Post**	**Pre**	**Post**	**Pre**	**Post**
10	E	74	22	Stroke	DNT	DNT	DNT	DNT	DNT	DNT	DNT	DNT
8	E	75	24	Stroke	68.1	65.4	60.0%	65.0%	16.7%	16.7%	82.7%	84.6%
47	E	54	8	Stroke	21.6	22.5	25.0%	25.0%	0.0%	1.7%	46.2%	67.3%
49	E	79	6	Stroke	DNT	DNT	DNT	DNT	DNT	DNT	DNT	DNT
4	E	56	141	Stroke	93	94.2	90.0%	100.0%	73.3%	80.0%	96.2%	98.1%
38	E	64	88	TBI	77.9	74.6	90.0%	80.0%	60.0%	53.3%	90.4%	86.5%
40	E	67	94	Stroke	73.7	79.5	50.0%	60.0%	58.3%	61.7%	96.2%	86.5%
48	E	66	129	Stroke	48.7	41.5	70.0%	60.0%	6.7%	3.3%	90.4%	94.2%
46	E	61	54	Stroke	91.2	81.76	95.0%	80.0%	95.0%	81.7%	96.2%	100.0%
24	E	58	109	Stroke	75.7	78.3	85.0%	90.0%	86.7%	95.0%	98.1%	98.1%
1	E	75	63	Stroke	64.4	80	65.0%	90.0%	46.7%	76.7%	82.7%	94.2%
12	E	47	44	Stroke	96.3	96.6	90.0%	95.0%	91.7%	90.0%	98.1%	96.2%
7	E	58	75	Stroke	80	88.9	55.0%	85.0%	56.7%	70.0%	96.2%	88.5%
2	E	67	60	Stroke	31.3	43.7	35.0%	65.0%	0.0%	8.3%	80.8%	84.6%
11	E	76	177	TBI	63.2	68.5	75.0%	80.0%	13.3%	36.7%	78.8%	78.8%
13	E	68	87	Stroke	70.5	64	75.0%	55.0%	45.0%	55.0%	90.4%	90.4%
35	E	59	72	Stroke	98.9	98.4	100.0%	100.0%	98.3%	95.0%	98.1%	94.2%
27	E	75	141	Stroke	93.4	95.1	55.0%	75.0%	80.0%	83.3%	88.5%	90.4%
18	E	71	46	Stroke	44.5	45	70.0%	70.0%	0.0%	0.0%	84.6%	86.5%
25	E	50	178	Stroke	59.3	60.4	50.0%	55.0%	65.0%	46.7%	90.4%	94.2%
20	E	68	23	Stroke	59	62.6	45.0%	60.0%	13.3%	26.7%	76.9%	76.9%
5	E	71	24	Stroke	42.4	45.7	50.0%	50.0%	0.0%	6.7%	65.4%	71.2%
14	E	50	33	Stroke	93.9	96.4	100.0%	100.0%	98.3%	DNT	98.1%	96.2%
6	E	72	22	Stroke	77.9	80.9	85.0%	85.0%	85.0%	83.3%	92.3%	96.2%
28	E	52	27	Stroke	90.2	89.5	70.0%	80.0%	68.3%	60.0%	78.8%	78.8%
17	E	66	14	Stroke	60.2	71.6	25.0%	35.0%	0.0%	3.3%	63.5%	65.4%
3	E	71	65	TBI	27.8	26.6	25.0%	35.0%	0.0%	0.0%	59.6%	51.9%
23	E	53	32	Stroke	91	91.6	70.0%	80.0%	51.7%	56.7%	94.2%	88.5%
9	E	38	16	Stroke	97.6	98.4	95.0%	100.0%	88.3%	96.7%	96.1%	100.0%
15	E	46	60	TBI	72	89.4	70.0%	90.0%	15.0%	18.3%	80.8%	75.0%
34	E	38	54	Stroke	77.7	77.1	85.0%	85.0%	55.0%	68.3%	96.2%	DNT
36	E	83	41	Stroke	90.7	89	95.0%	100.0%	90.0%	DNT	96.2%	96.2%
43	E	66	31	Stroke	89.9	95.6	90.0%	95.0%	73.3%	63.3%	86.5%	92.3%
33	E	74	15	Stroke	49.7	72.3	55.0%	65.0%	28.3%	13.3%	75.0%	84.6%
22	E	74	12	Stroke	67.6	81.5	65.0%	65.0%	91.7%	90.0%	92.3%	96.2%
42	E	55	11	Stroke	93.9	96.1	100.0%	100.0%	100.0%	98.3%	98.1%	98.1%
21	E	72	1	Stroke	11.5	41.3	60.0%	DNT	5.0%	10.0%	82.7%	94.2%
32	E	56	2	Stroke	97.2	96.6	85.0%	90.0%	83.3%	71.7%	92.3%	94.2%
26	E	70	14	Stroke	15.6	23.1	60.0%	70.0%	0.0%	0.0%	73.1%	73.1%
44	E	67	4	Stroke	99.9	100	95.0%	95.0%	98.3%	96.7%	96.2%	96.2%
45	E	66	18	Stroke	20.8	33	65.0%	65.0%	0.0%	1.7%	84.6%	90.4%
51	E	64	23	Stroke	75.9	77.7	85.0%	80.0%	41.7%	45.0%	98.1%	DNT
**Avg**.	**E**	**51.4**	**64**		**68.9**	**72.9**	**70.3%**	**75.8%**	**49.5%**	**49.1%**	**86.5%**	**87.6%**
**SD**	**E**	**47.0**	**11**		**25.7**	**23.2**	**21.6%**	**19.6%**	**37.2%**	**35.4%**	**12.1%**	**11%**
50	C	60	46	Stroke	93.7	97.7	40.0%	75.0%	93.3%	95.0%	90.4%	94.2%
16	C	71	78	Stroke	12	8.5	30.0%	35.0%	0.0%	0.0%	67.3%	65.4%
41	C	53	285	Stroke	71.3	72.8	55.0%	60.0%	38.3%	41.7%	78.8%	88.5%
39	C	58	359	TBI	83.2	85.2	85.0%	60.0%	25.0%	26.7%	84.6%	84.6%
19	C	87	13	Stroke	88.7	91.3	65.0%	65.0%	58.3%	56.7%	DNT	80.8%
37	C	65	29	Stroke	27.9	24.9	55.0%	55.0%	0.0%	0.0%	84.6%	94.2%
29	C	68	21	Stroke	95	94.4	95.0%	85.0%	90.0%	78.3%	96.2%	96.2%
30	C	68	22	Stroke	44.1	48.5	25.0%	25.0%	1.7%	0.0%	21.2%	55.8%
31	C	74	29	Stroke	93.7	96.4	70.0%	75.0%	93.3%	95.0%	82.7%	90.4%
**Avg**.	**C**	**98**	**67.1**		**67.7**	**68.9**	**57.8%**	**59.4%**	**44.4%**	**43.7%**	**75.7%**	**83.3%**
**SD**	**C**	**129.7**	**10.0**		**31.7**	**33.6**	**23.7%**	**19.3%**	**40.7%**	**39.8%**	**23.6%**	**14%**

*Reported severity scores include R-WAB AQ, CLQT Composite Severity scores, BNT, and PAPT*.

*E, Experimental group; C, Control group, and DNT, Did Not Test. Two participants did not complete any testing due to inability and unwillingness to complete tests*.

**Figure 2:**
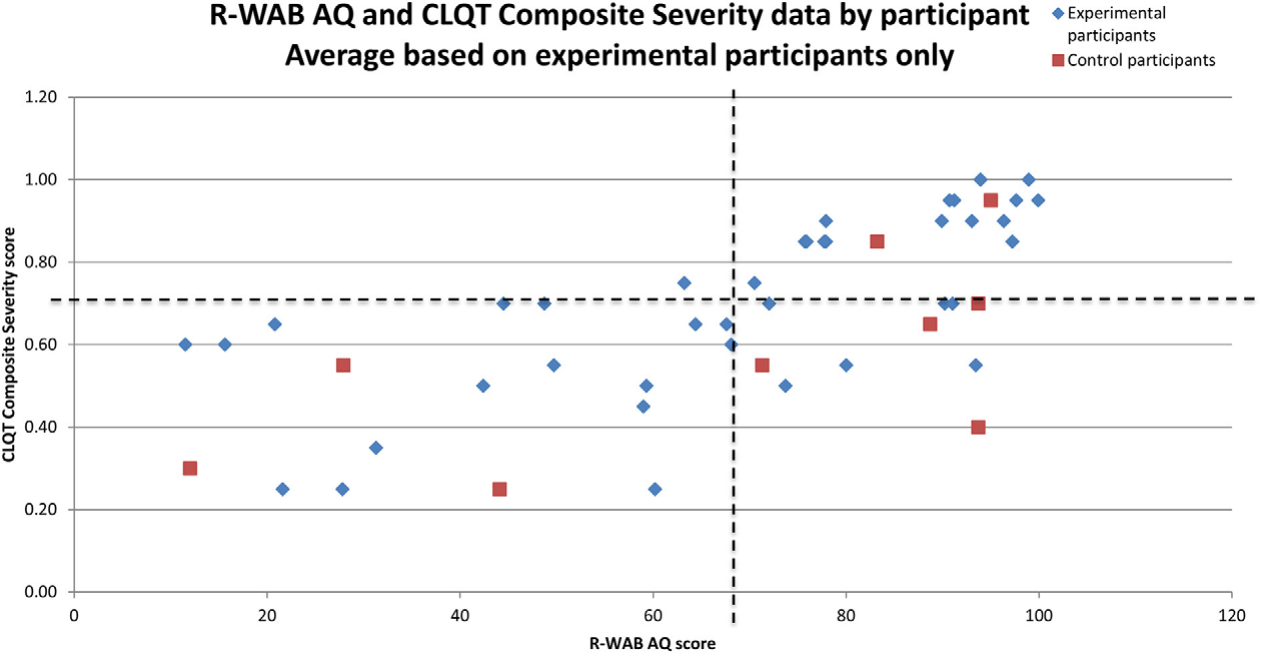
**Scatter plot of low vs. high deficits in R-WAB AQ (x-axis) and CLQT Composite Severity (y-axis) by patient**. The dotted lines denote the average R-WAB AQ and average CLQT Composite Severity score for experimental participants to provide more information for **Table 3B**.

Participants were administered standard language and cognitive tests prior to and following the completion of a 10 week therapy program: *Revised-Western Aphasia Battery* (R-WAB, Kertesz, 1982), *Cognitive Linguistic Quick Test* (CLQT, Helm-Estabrooks, 2001), *Boston Naming Test* (BNT, Goodglass et al., 1983), and *Pyramids and Palm Trees* (PAPT, Howard and Patterson, 1992). Refer to Table 1 for all participants' scores from pre- and post-therapy for several of these tests and subtests. The *R-WAB* was used to determine the type and level of aphasia severity; the mean pre-therapy scores on the *Aphasia Quotient* (AQ) for the experimental group (*M* = 68.9, *SD* = 25.7) was not significantly different from the control group (*M* = 67.7, *SD* = 31.7) (*t* = 1.23, *p* = 0.36). The *R-WAB* also provided the *Cortical Quotient* (CQ) of each participant and the mean pre-therapy scores for the experimental group (*M* = 69.5, *SD* = 24.5) was not significantly different from the control group (*M* = 64.9, *SD* = 27.8) (*t* = 0.85, *p* = 0.42). The *CLQT* was used to determine the relative contribution of cognitive deficits to language dysfunction and to rule out dementia. Pre-therapy Composite Severity scores for experimental participants (*M* = 2.8/4, *SD* = 0.9) was not significantly different from the control group (*M* = 2.3/4, *SD* = 0.9) (*t* = 1.10, *p* = 0.64). Refer to Figure 2 for a scatterplot of all participants' *R-WAB AQ* and *CLQT* Composite Severity scores from pre-therapy testing. The *BNT* was used to determine confrontation naming ability; mean pre-therapy scores for the experimental participants (*M* = 31.1/60, *SD* = 22.2) was not significantly different from the control group (*M* = 26.7/60, *SD* = 24.4) (*t* = 1.09, *p* = 0.65). The *PAPT* was used to test the participants' semantic access and mean pre-therapy scores for experimental participants (*M* = 45.1/52, *SD* = 6.2) was significantly different from the control group (*M* = 39.4/52, *SD* = 12.3) (*t* = 1.94, *p* < 0.01).

### Stimuli

All therapy tasks were implemented on an iPad using the Constant Therapy iOS platform (www.constanttherapy.com). Details regarding task creation are described later. The tasks were designed with simple visual configurations and utilized similar methods of response. For each item, instructions were provided both visually and auditorily. Auditory instructions and stimuli could be repeated any number of times. For tasks that contained images, the stimuli could be enlarged to the entire screen of the iPad. Several tasks also included buttons that revealed a hint to answering the item (e.g., Rhyming instructions: “Does [the pictured object] rhyme with pin?” Auditory hint: “Pig”). Participants could either answer the item or, if stuck, skip the item, which would reveal the answer and allow them to move onto the next item. At the completion of every item, participants were told visually and auditorily if their response was correct or incorrect and the correct answer was available for review. Every button on the interface that participants could interact with was tracked by the software.

Thirty-seven therapy tasks were developed based on a review of evidence based therapy recommendations from various sources, including Speechbite (http://speechbite.com/), PubMed (National Center for Biotechnology Information, U.S. National Library of Medicine, Bethesda, MD), and Google Scholar (Google Inc., Mountain View, CA) (refer to Supplementary Table 1 for descriptions and specific evidence for the therapy tasks). The tasks were divided into a hierarchy and classified as either language or cognitive tasks; language therapy tasks were divided into (1) Naming therapy: (a) Rhyming, (b) Syllable Identification, (c) Sound Identification, (d) Category Matching, (e) Feature Matching and (f) Picture Naming; (2) Reading therapy: (a) Word Identification, (b) Category Identification, (c) Reading Passage, (d) Long Reading Comprehension, and (e) Map Reading, (3) Writing therapy: (a) Word Copy Completion, (b) Word Copy, (c) Word Spelling Completion, (d) Word Spelling, (e) Picture Spelling Completion, (f) Picture Spelling, (g) Sound to Letter Matching, and (h) Letter to Sound Matching; and (4) Sentence planning: (a) Active Sentence Completion and (b) Passive Sentence Completion. Cognitive therapy tasks were divided into (1) Visuo-spatial processing: (a) Symbol Matching, (b) Clock Reading; (2) Memory: (a) Picture Matching, (b) Word Matching, (c) Sound Matching, and (d) Voicemail; (3) Attention: (a) Flanker and (b) Symbol Matching; (4) Problem solving: (a) Analytical reasoning, which further included: (i) Word Ordering and (ii) Picture Ordering, (b) Arithmetic, which included: (i) Addition, (ii) Subtraction, (iii) Multiplication, and (iv) Division, and (c) Quantitative reasoning, which included: (i) Clock Math and (ii) Word Problems; and (5) Executive function: (a) Instruction Sequencing.

All naming and writing tasks used stimuli items that were developed from a database of Mturk tasks. Several other tasks also utilized this database of stimuli, including Word Identification, Category Identification, Word/Picture/Sound Matching, and Word/Picture Ordering. The rest of the tasks were built from scratch, with individual databases for each task. Full details regarding the tasks and stimuli are out of the scope of this paper, but Supplementary Table 1 includes additional information such as descriptions of the tasks and the number of items per task. All auditory stimuli and instructions were recorded using a Blue Icicle USB Microphone Converter. They were recorded and edited with Audacity software using both male and female voices. Visual stimuli consisted of colored pictures taken from various image databases and from public domain. Because the software selected items from the database in a random order, so every item the participants saw in a given session was unique, though some items may have been repeated over the 10 week period, which varied based on the size of the database for the task.

### Design

The general procedures of the therapy are described by Kiran et al. (2014). Prior to initiating therapy, all participants were oriented to using the iPad and the therapy application. If the experimental participants already owned an iPad, the Constant Therapy software was downloaded onto it; if not, they were loaned an iPad2 for the duration of the therapy. Caregivers for the experimental participants were encouraged to sit in on this session so they could assist the participant with the iPad while at home. More information on the steps involved in the therapy is described below.

#### Assess

Prior to the initial session with the iPad, the clinician would assess the strengths and weaknesses of each participant based on their language and cognitive profile as a result of performance on the standardized tests. From that profile, a set of potential tasks were selected for each participant to complete during the initial session. For example, if a participant scored 50% on Spoken Word—Written Word Matching in the Reading portion of the *R-WAB*, the clinician would assess the participant's ability on Word Identification.

#### Assign

Generally, if the participant scored below 80% during the first assessment of the task (or baseline) the clinician would assign ^1^ the task to the participant's schedule^2^ for continued practice. If participants performed very low (lower than 40%) on a task, they were assigned a lower level of that task or a task examining a similar skill. Because both accuracy and latency were examined as dependent measures, 80% accuracy was chosen as a cutoff to allow for examination of a maximum of 20% change in accuracy and corresponding decreases in latency. In most cases, if a participant performed above 80% on the baseline task, the task was not assessed further; instead the next level of difficulty of that task was assessed or a different task examining the same skill was assessed (e.g., Rhyming instead of Sound Identification). In some cases, a task was still assigned when accuracy was above 80% on the baseline if the latency was high or if the participant requested to work on the task further.

Throughout the therapy, there were two types of sessions: sessions completed in the clinic (assisted sessions) and sessions completed as homework (scheduled sessions). The order of the tasks and the number of items practiced were set by the clinician for both types of sessions. During an assisted session, the clinician controlled the flow of the tasks; each task was assessed one at a time and a report of how the participant performed was available at the completion of each task. Further, participants were given more one-on-one feedback compared to scheduled sessions, where automated feedback for each item was provided by the software. The tasks assigned to the experimental participants for homework depended on how the participant performed during the clinic sessions. During a scheduled session, participants practiced all of the homework tasks in a row.

#### Act

**Figure 3:**
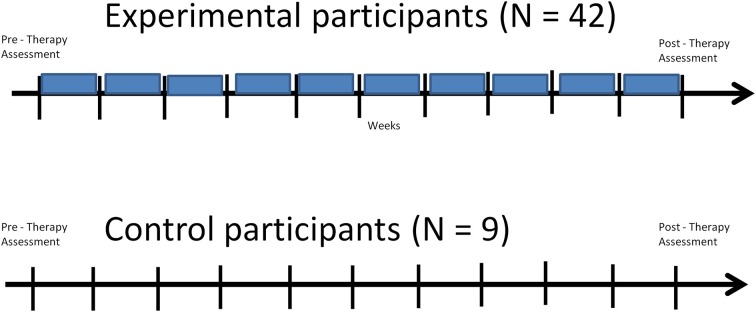
**Structure of Experimental Design for experimental and control participants: both groups receive the same pre- and post-therapy assessments and the same number of clinic sessions throughout the therapy (denoted by the black vertical lines), but experimental participants additionally receive scheduled sessions while at home, which take place in between the clinic sessions throughout the therapy program (denoted by the blue boxes)**.

The third part of the process is when the participants actually practiced the therapy. The control group's therapy consisted only of 10 weekly assisted sessions. Experimental participants had both assisted and scheduled sessions (refer to Figure 3 for a view of the experimental design). Refer to Tables 2A,C for language task assignment by participant and to Tables 2B,D for cognitive task assignment by participant. The tables depict how the therapy was individualized for each participant; for instance participant 10 was assessed on and assigned only five tasks during the 10 week therapy, while participant 12 was assessed on 22 tasks and assigned 14 tasks.

**Table 2A:**
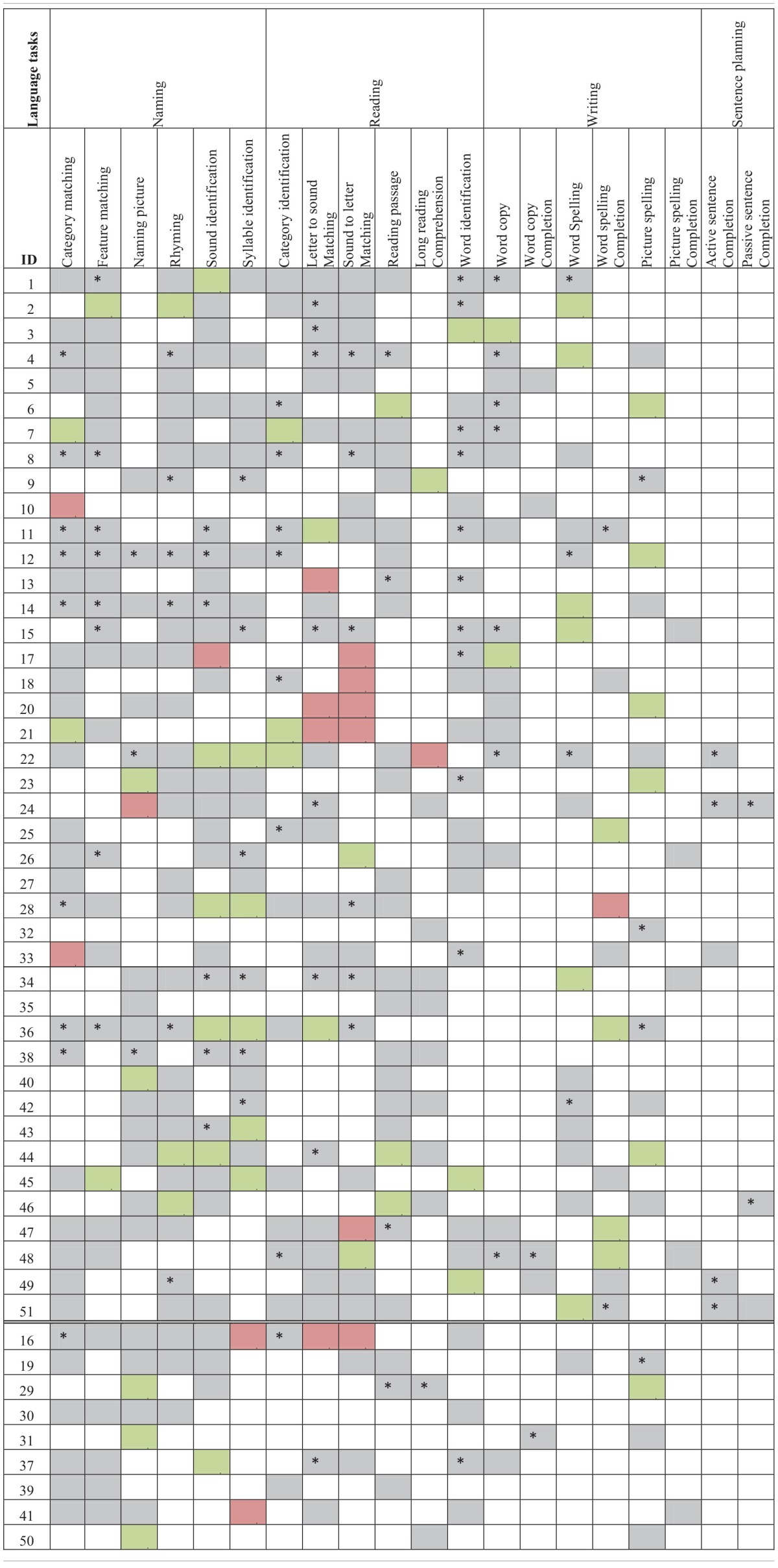
**Language task assignment by participant with significant accuracy slope analysis results^4^**.

All highlighted cells were tasks that were assessed, and of those cells, tasks highlighted in any color are the slope coefficient values found to be significant in the individual regression analysis (see Results for details). Cells highlighted in green denote slope coefficient values from the regression considered to be beneficial (increases in accuracy per session). Cells highlighted in red denote slope coefficient values from the regression considered to be non-beneficial (decreases in accuracy per session). ^*^Denotes the task was only assessed once.

**Table 2B:**
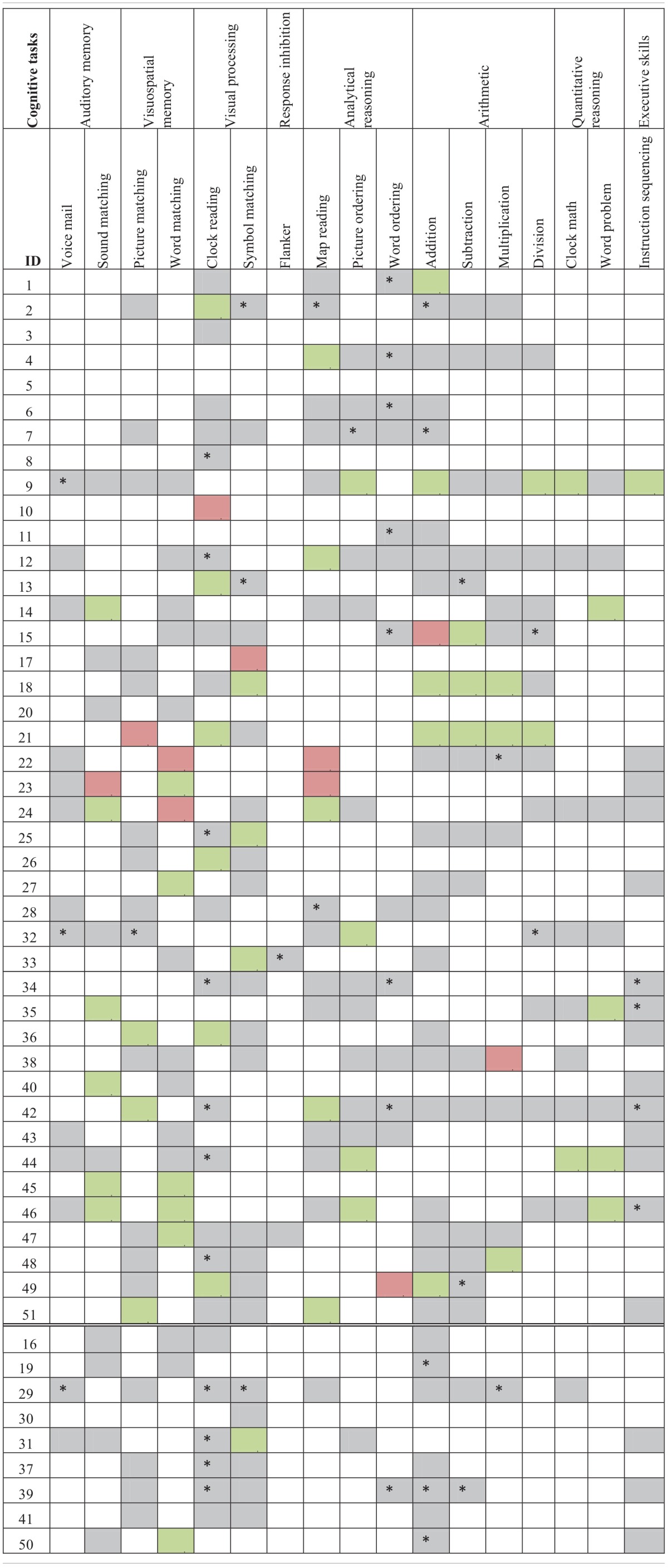
**Cognitive task assignment by participant with significant accuracy slope analysis results**.

All highlighted cells were tasks that were assessed, and of those cells, tasks highlighted in any color are the slope coefficient values found to be significant in the individual regression analysis (see Results for details). Cells highlighted in green denote slope coefficient values from the regression considered to be beneficial (increases in accuracy per session). Cells highlighted in red denote slope coefficient values from the regression considered to be non-beneficial (decreases in accuracy per session). ^*^Denotes the task was only assessed once.

**Table 2C:**
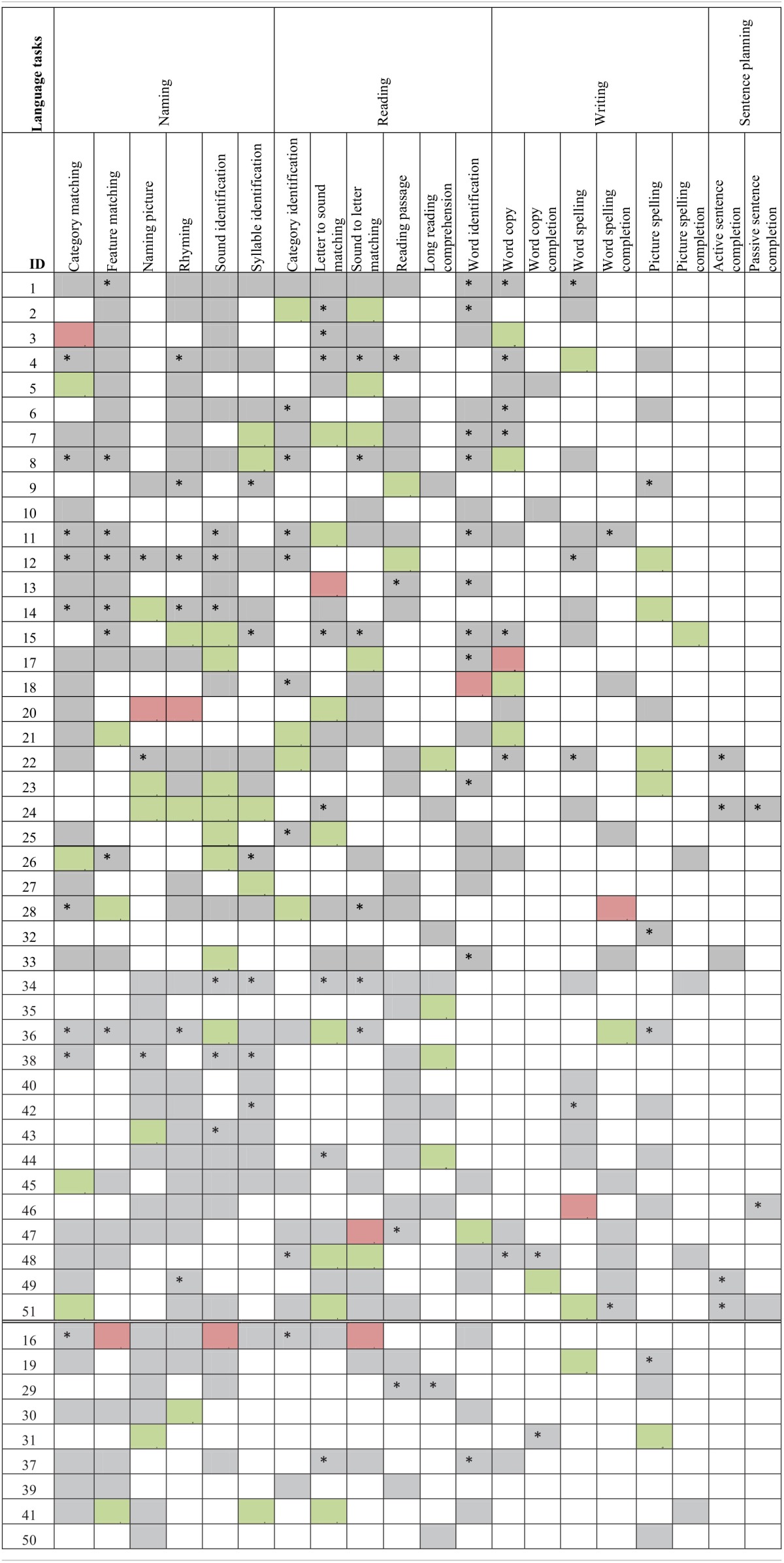
**Language task assignment by participant with significant latency slope analysis results**.

All highlighted cells were tasks that were assessed, and of those cells, tasks highlighted in any color are slope coefficient values found to be significant in the individual regression analysis. Cells highlighted in green denote slope coefficient values from the regression considered to be beneficial (decreases in latency per session). Cells highlighted in red denote slope coefficient values from the regression considered to be non-beneficial (increases in latency per session). ^*^Denotes the task was only assessed once.

**Table 2D:**
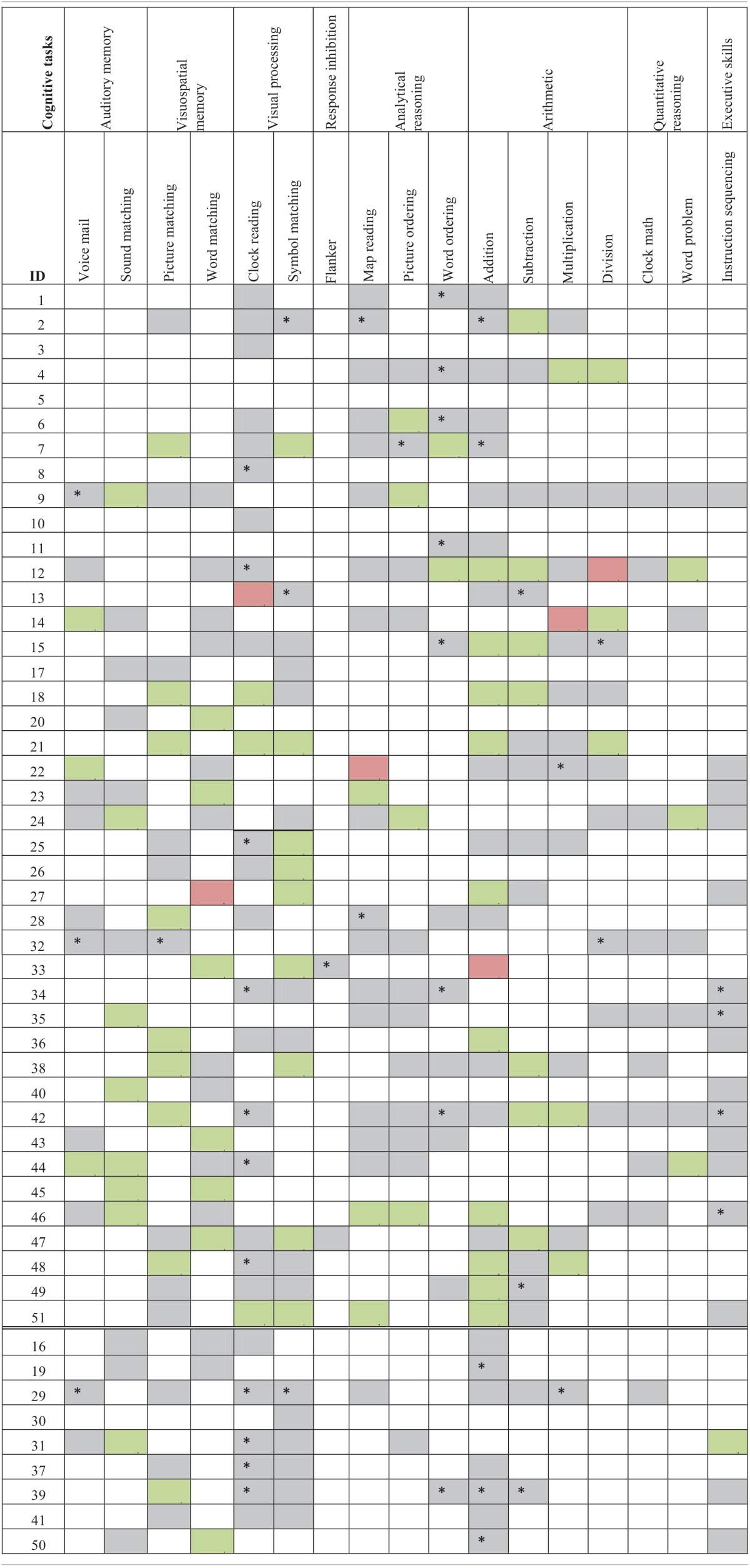
**Cognitive task assignment by participant with significant latency slope analysis results**.

All highlighted cells were tasks that were assessed, and of those cells, tasks highlighted in color are slope coefficient values found to be significant in the individual regression analysis. Cells highlighted in green denote slope coefficient values from the regression considered to be beneficial (decreases in latency per session). Cells highlighted in red denote slope coefficient values from the regression considered to be non-beneficial (increases in latency per session). ^*^Denotes the task was only assessed once.

#### Analyze

All participants' performances were analyzed on a real-time basis, using both clinic sessions and reports located on the application (see below). Each task was practiced until accuracy reached 95% or higher on two or more occasions, at which point the clinician would either assess the next level of difficulty or they would remove that task from the participant's schedule and would assess a new task; this assessment and alteration of schedules could be completed either during assisted sessions or remotely during the week for experimental participants. For example, a participant was working on level one of Word Spelling (which consists of two to four letter words) and achieved 95% accuracy during their session on Monday and then 98% accuracy during their session on Thursday. The clinician would remove level one from the participant's schedule and then assess level two of Word Spelling (which consists of five or six letter words). In this way, participants progressed through tasks with increasing difficulty in a personalized, self-paced manner. Additionally, if a participant was not improving on a task over time, either a lower level of that task was assigned in addition to or in replacement of the original task, or a different task examining the same skill (e.g., Word Spelling instead of Picture Spelling) was assigned.

**Figure 4:**
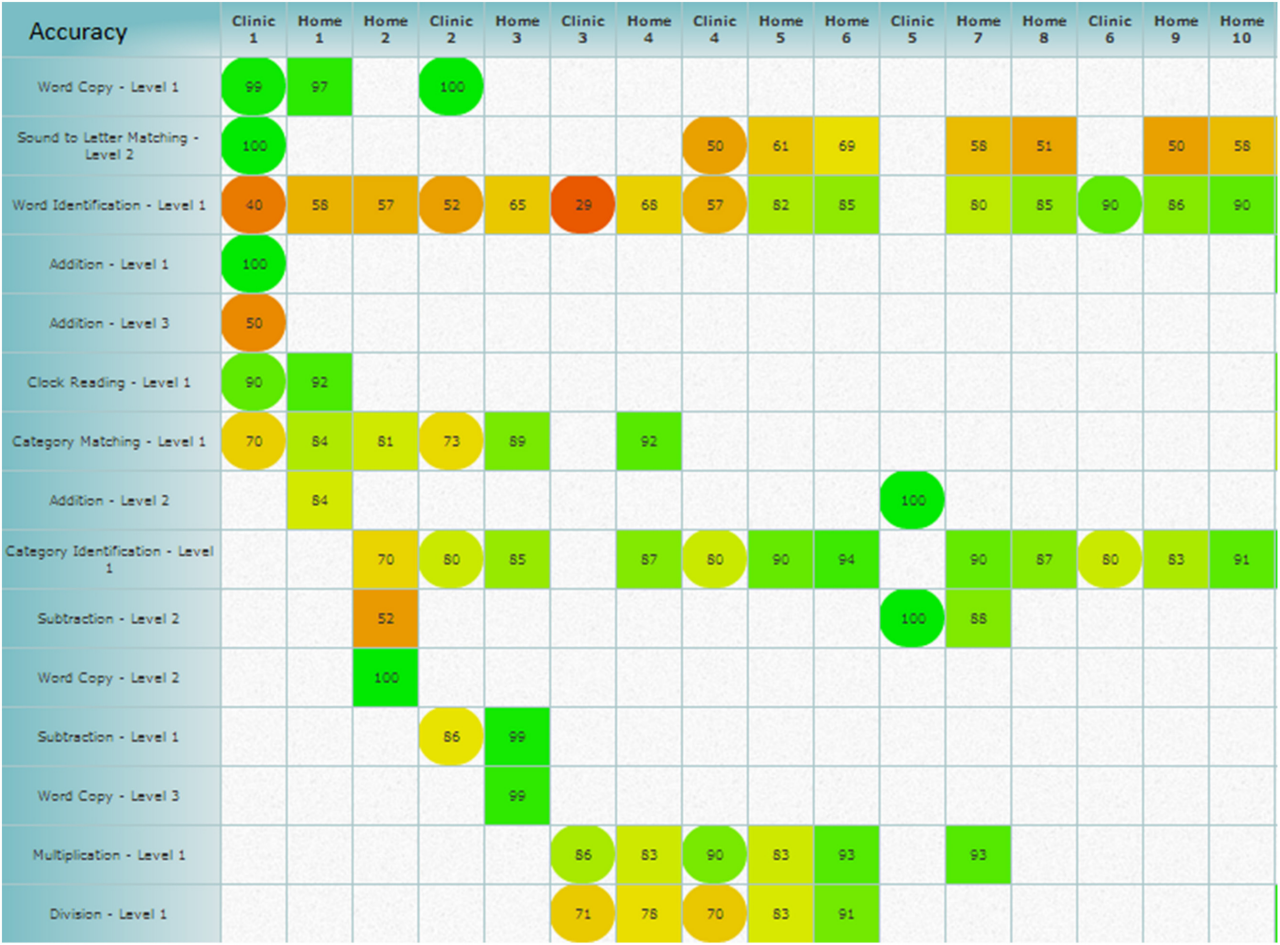
**Weekly schedule analysis provided by the application, which shows the averaged accuracy and could also show averaged latency scores by week**. Square cells denote scheduled sessions and circle cells denote assisted sessions, while the coloring gives information on how the averaged accuracy score falls on the scale of zero (red) to 100% (green). Figure obtained using Constant Therapy, www.constanttherapy.com.

In addition to real-time analysis, all participants' performances were analyzed after completion of the therapy. The Constant Therapy software generated reports for each participant, which included averaged accuracy and latency ^3^ for every session, specific to each level of all tasks the participants completed. Reports of each participant's data, including tasks they completed three or more times, could be analyzed three different ways, (a) latency and accuracy by week, (b) overall accuracy and latency for each task averaged by session, and (c) accuracy and latency by individual item. Specifically, the application displayed task accuracy and latency by week, this information was used to personalize the therapy progression for each individual (Figure 4). Second, the data could be analyzed by each level of every task the participants completed, averaged by session, which was later used to complete further analysis of each task. Finally, the data could be analyzed by individual items completed in each level of every task by participant. These analyses have not been completed yet due to the large amounts of data involved. Also included in the session reports was information on whether the session was assisted or scheduled as well as how many items were completed by the participants in each session. From these session reports, several results could be calculated, including total therapy duration in weeks and number of clinic sessions (*dosage*) and the amount of time spent on the therapy (*compliance*).

For all the accuracy and latency data discussed in the results, only tasks that included more than two participants that had completed three or more sessions were entered into the analysis. Also, tasks that had multiple levels of difficulty were treated no different from tasks with only one level due to the scope of this paper. Thus, all tasks are collapsed across levels. Statistical analyses on the data ranged from (a) correlations to examine how dosage and compliance affect improvement on the therapy tasks (Q1) (b) linear mixed effect models to examine the effectiveness of the therapy (Q2) and how individual standardized scores factored into the effectiveness of the therapy (Q3), (c) individualized regression analyses to examine individual performance on the tasks (Q3) and to examine co-improvement between tasks (Q4), and (d) paired *t*-tests to examine changes on standardized tests for the experimental and control groups (Q5). All analyses were completed in Statistical Analysis Software (SAS Institute Inc., Cary, NC), Statistical Package for the Social Sciences (SPSS Inc., Chicago, IL), Statistica software (StataCorp, College Station, TX), and the statistical software package “R” (R Foundation for Statistical Computing, Vienna, Austria).

## Results

The following results are broken down into sub-sections that examine different types of results: usage data and duration of therapy, task effectiveness, individual participant performance in therapy, co-improvement between tasks, and participants' changes on standardized measures.

### Therapy dosage and therapy compliance

**Figure 5:**
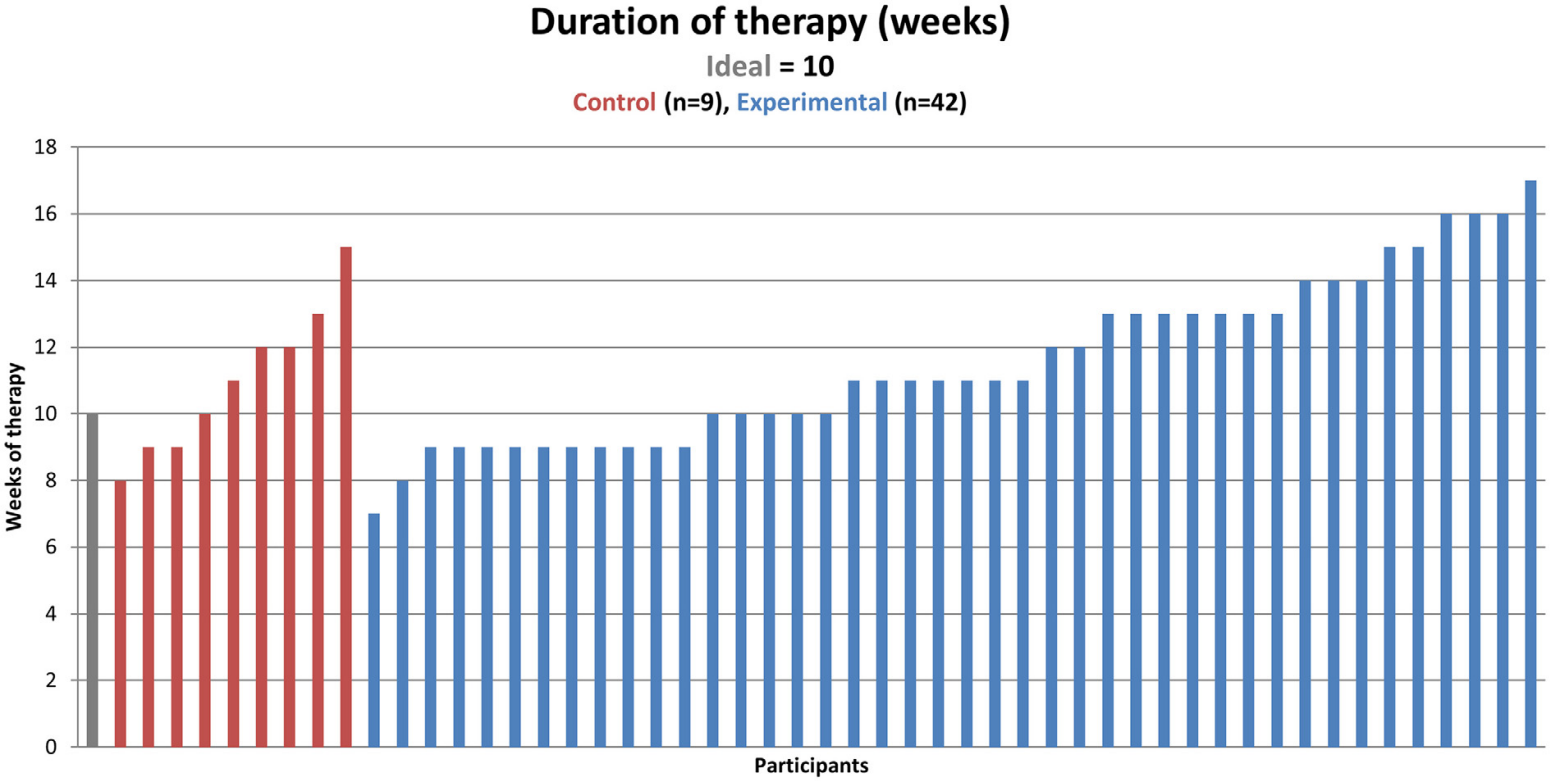
**Duration of therapy in calendar weeks (one measure of dosage, represented on the y-axis) by participant (found on the x-axis)**. The ideal number of weeks is 10 for both groups, but due to some participants finishing before 10 clinic sessions, some participants were in the therapy for fewer than 10 weeks, and due to missed and make-up sessions, some participants were in the therapy for more than 10 weeks.

Participants in the experimental group were assigned between two and 11 (*M* = 3) tasks at a given time. Control participants completed between two and 14 tasks during each weekly session in the clinic (*M* = 3). Experimental and control participants did not differ in terms of the total clinic sessions: control participants had an average of 9.67 clinic sessions (*SD* = 1) while experimental participants had an average of 9.60 clinic sessions (*SD* = 1.4), which was non-significant (*t* = 1.36, *p* = 0.36)^5^. A measure of calendar weeks that the therapy lasted was also calculated since this did not always match the number of clinic sessions the participants completed. This was an especially important to measure because experimental participants practiced while away from the clinic. The two groups did not differ in terms of the duration of therapy: control participants had an average of 11 weeks (*SD* = 2.2) in therapy and experimental participants had an average 11.5 weeks (*SD* = 2.5) in therapy (*t* = 1.12, *p* = 0.78, Figure 5)^6^. Therefore, there was no difference in therapy *dosage* either measured by the number of clinic sessions or the number of weeks of therapy.

**Figure 6:**
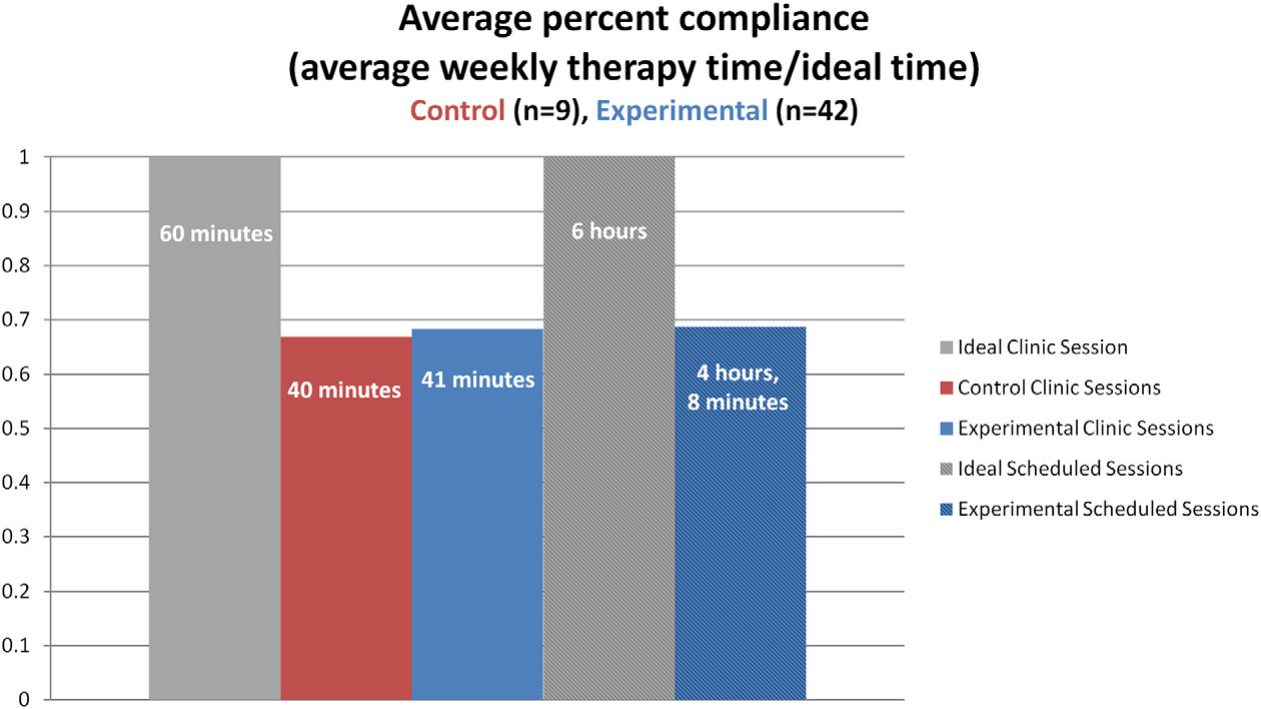
**Average weekly percent of compliance (percent compliance, represented on the y-axis) by group and type of session (found on the x-axis)**. The ideal time for an assisted session is 1 h while the ideal time for weekly scheduled home practice is 6 h (six scheduled sessions lasting 1 h each for the remaining days of the week).

Next, *compliance* was calculated based on how much time the participants spent on the tasks. This differed greatly between control and experimental participants, which was expected due to the extra scheduled sessions experimental participants completed. Therefore, an ideal control participant would practice the therapy for 1 h each week and an ideal experimental participant would practice for 1 h each week in the clinic sessions and for 6 h each week while at home. In fact, on average, control participants worked on the application for 40 min during each assisted session (*SD* = 7 min), which is 66.9% compliance out of the ideal therapy time. The experimental participants worked on the therapy for 41 min at the clinic each week (*SD* = 14 min), which is 68.3% compliance out of the ideal therapy time and then practiced at home for 4 h and 8 min each week (*SD* = 3 h and 25 min), which is 68.7% compliance out of the ideal therapy time (see Figure 6). Correlations using pair-wise deletion of missing cases were completed to examine how individual differences (age, *MPO*, initial *R-WAB AQ* scores, and initial *CLQT* Composite Severity scores) related with compliance. Results showed that only initial *R-WAB AQ* scores significantly correlated with percent compliance for scheduled sessions (*r* = −0.33, *p* < 0.05). To test for a difference in compliance percentage for both assisted and scheduled session between injury types, all *TBI* participants were matched with a subgoup of stroke participants by age, *MPO*, group (experimental or control), and gender (when possible). Independent *t*-tests were completed for the matched pairs of participants, neither of which were significant (assisted sessions: *t* = 0.10, *p* = 0.92, scheduled sessions: *t* = 1.15, *p* = 0.29).

### Task effectiveness

To examine the effectiveness of the language and cognitive therapy tasks, two questions were asked. First, is there a significant difference in improvement between the experimental and control groups? Second, is there significant improvement in outcomes for the experimental group over the course of the therapy across tasks? To answer these questions, two linear mixed effects models were built with log odds of accuracy as the dependent measure and two models with log odds of latency as the dependent measure to examine the effect of time (in days) on therapy outcome while controlling for severity scores. Estimates generated from the mixed model analysis show the average daily change per session of each outcome for each task significant at *p* < 0.05. For accuracy, improvement is positive daily change while for latency it is negative. Given the number of results, the discussion of what the estimate values mean is out of the scope of this paper.

Table 3A contains the results of the task specific mixed model analysis for both experimental and control participants for only assisted sessions with time as a factor. In terms of accuracy, there were no tasks on which both groups showed overall therapy improvement. However, experimental participants showed improvements in accuracy on Rhyming and Syllable Identification, Word Matching, and Word Problem. Control participants only showed improvements in accuracy on Naming Picture. Accuracy on Word Spelling Completion significantly decreased as a function of therapy for the experimental group, while the control group showed a decrease on Syllable Identification and Word Spelling. When examining latency, overall therapy gains were observed for both groups on Category Matching. Experimental participants also showed improvements in latency (faster response times) on Rhyming, Sound Identification, Category Identification, Word Matching, and Multiplication. Control participants showed improvements in latency on Voice Mail and Instruction Sequencing. Latency significantly decreased (slower response times) for the experimental group on Word Spelling Completion and Symbol Matching, while the control group showed no significant decreases in latency.

**Table 3A:**
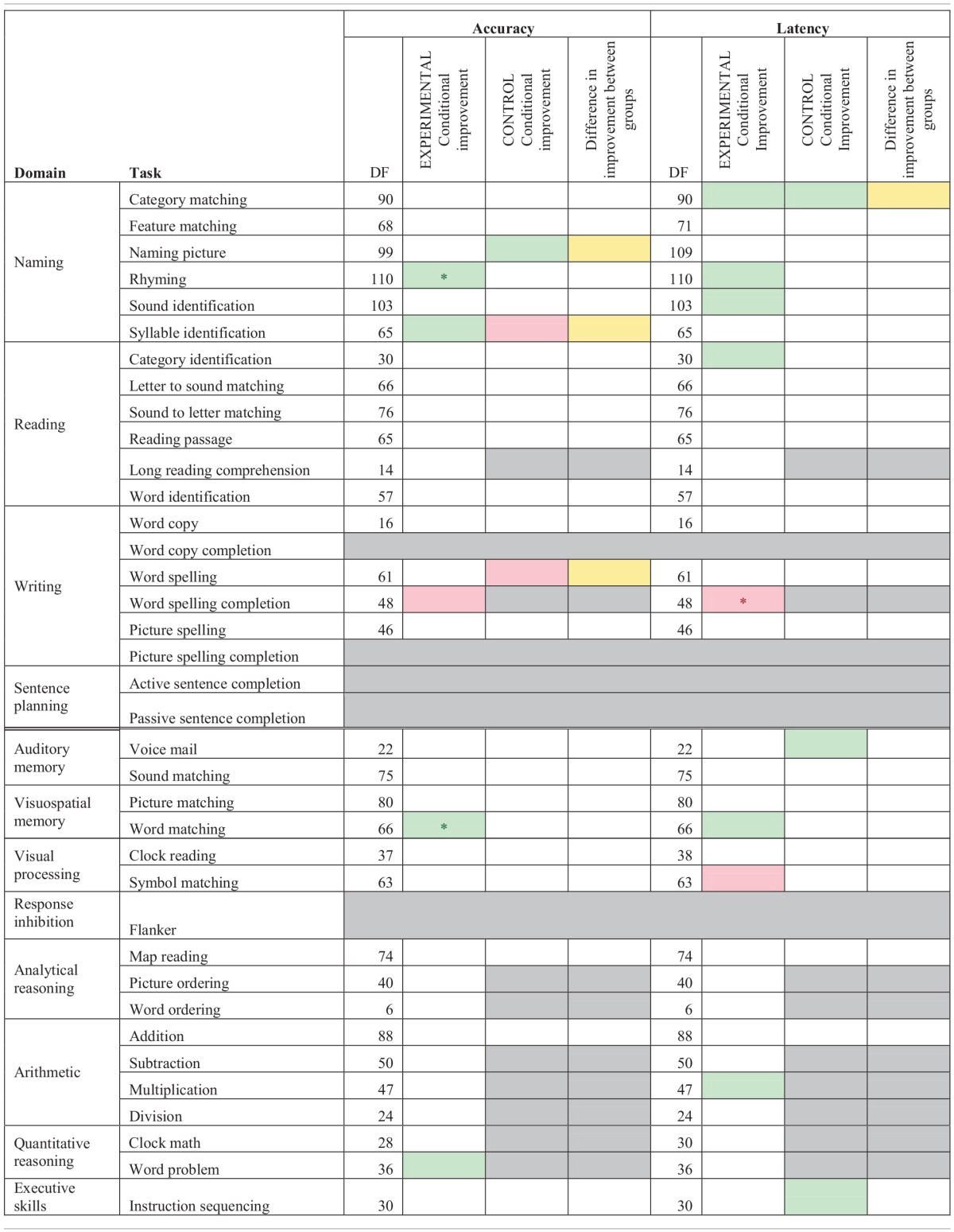
**Accuracy and latency estimate of log odd change per session for mixed model analysis of assisted sessions for experimental and control participants by task, while controlling for severity scores**.

When the experimental conditional improvement is highlighted it means that overall, experimental participants show significant change on that task and when control conditional improvement is highlighted it means that overall, control participants show significant change on that task. For both experimental and control conditional improvements, green highlights mean the change is considered beneficial, and pink highlights mean the change is considered non-beneficial. When the difference in improvement between groups is highlighted, it means that there is a significant difference between the conditional improvements of the two groups. Gray highlighting means that there was not enough data from the participants to include in the analysis. A ^*^ denotes significance at 0.06 while all other colored cells are significant at the 0.05 level.

Significant differences between the two groups in terms of accuracy on the assisted sessions (see Table 3A) arose for Syllable Identification and Word Spelling where experimental participants improved more than control participants while Naming Picture showed a significant difference between the groups where control participants improved more than experimental participants. Significant differences between the two groups on latency emerged for Category Matching where control participants showed a stronger significant improvement than experimental participants. Since this analysis is only looking at assisted sessions, the results are limited in their scope, but interestingly, the experimental group showed more changes than the control group.

Since there were more changes and larger amounts of data for the experimental group, the data from just the experimental participants was further examined for both assisted and scheduled sessions by task (refer to Table 3B). In the conditional improvement columns of the table, the overall effectiveness of a particular task was examined. A significant positive conditional improvement for a task means that the participants improved, whereas a significant negative conditional improvement for a task means that the participants declined on the task. Overall significant improvements in accuracy were seen for Category Matching, Feature Matching, Rhyming, Word Identification, Word Spelling, and Word Matching. Several significant overall decreases in accuracy were shown for Letter to Sound Matching, Long Reading Comprehension, and Addition. With regards to latency, significantly improved latency (faster reaction times) was observed for Feature Matching, Sound Identification, Letter to Sound Matching, Voice Mail, Sound Matching, and Word Matching. Significant decreases in latency (slower reaction times) were observed for Reading Passage and Word Identification, Picture Spelling, Addition, Clock Math, and Instruction Sequencing.

**Table 3B:**
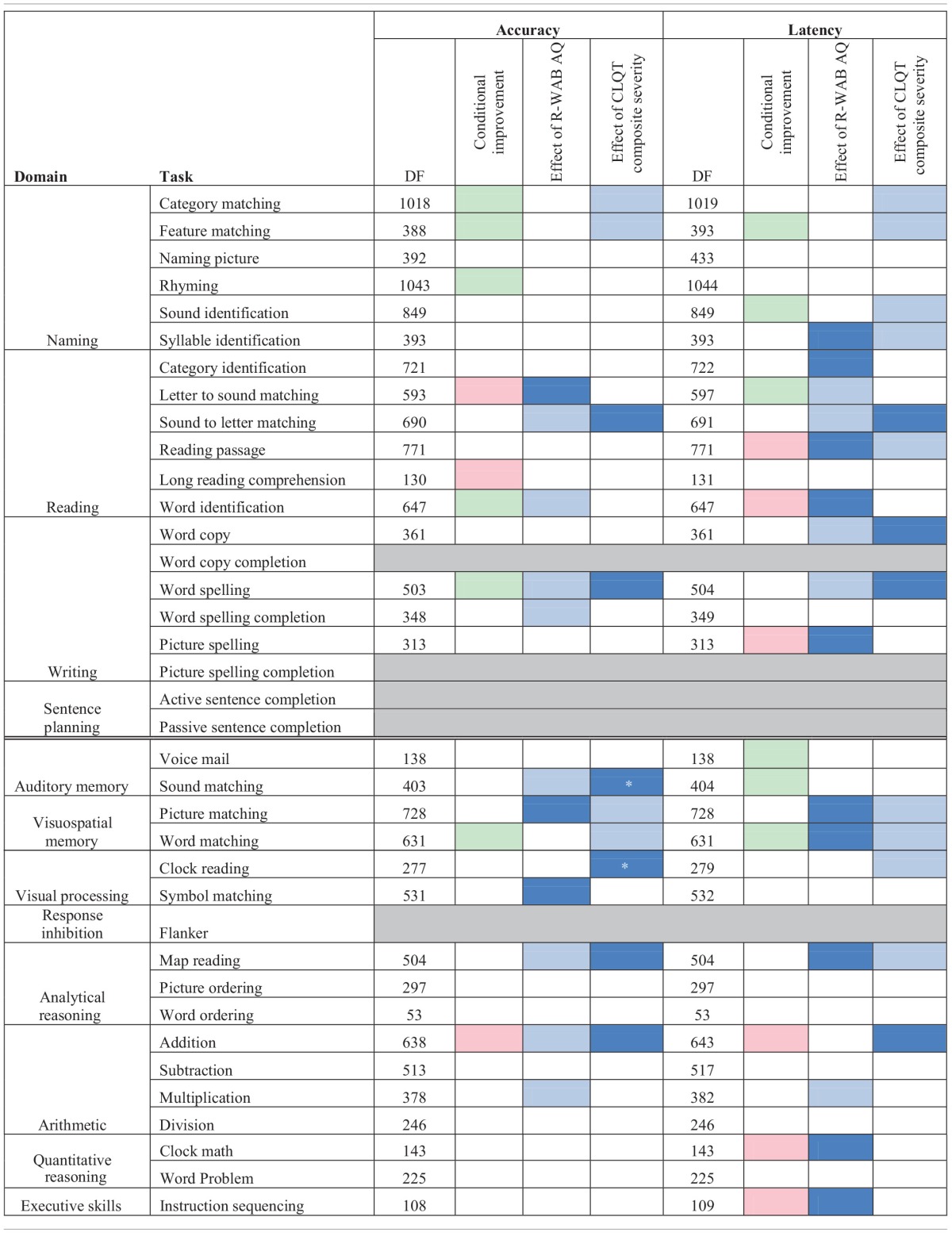
**Accuracy and latency estimate of log odd change per session for mixed model analysis of assisted and scheduled sessions for experimental participants by task**.

When the conditional improvement is highlighted in green, it means that overall, experimental participants show beneficial and significant change on that task and when the conditional improvement is highlighted in pink, it means that overall, experimental participants show non-beneficial and significant change on that task, controlling for R-WAB AQ and CLQT Composite Severity. When the effect of R-WAB AQ or CLQT Composite Severity is highlighted in dark blue it means that participants with a higher score than average show more improvement in the task, and when the effect of R-WAB AQ or CLQT Composite Severity is highlighted in light blue it means that participants with a lower score than average show more improvement in the task. Gray highlighting means that there was not enough data from the participants to include in the analysis. A ^*^ denotes significance at 0.06 while all other colored cells are significant at the 0.05 level.

### Individual responsiveness to therapy

Because overall improvements only indicate if there is a general effect of therapy, the influence of individual variability (i.e., participants' severity) on therapy outcomes was examined. Specifically, the effects of *R-WAB AQ* scores and *CLQT* Composite Severity on improvement were examined (see Table 3B). A positive effect of *R-WAB AQ* means that participants with higher *AQ* scores than average (refer to experimental participants who fall to the right of the vertical dotted line in Figure 2) showed more improvement on the task. A significant positive effect of *AQ* was observed in accuracy for Letter to Sound Matching, Picture Matching, and Symbol Matching and was observed in latency for Syllable Identification, Category Identification, Reading Passage, Word Identification, Picture Spelling, Picture Matching, Word Matching, Map Reading, Clock Math, and Instruction Sequencing. A negative effect of *AQ* means that participants with lower *AQ* scores than average (refer to experimental participants who fall to the left of the vertical dotted line in Figure 2) showed more improvement on the task. Significant negative effects were observed for accuracy in Sound to Letter Matching, Word Identification, Word Spelling, Word Spelling Completion, Sound Matching, Map Reading, Addition, and Multiplication were observed for Negative latency in Letter to Sound Matching, Sound to Letter Matching, Word Copy, Word Spelling, and Multiplication.

A positive effect of *CLQT* Composite Severity means that participants with higher than average Composite Severity scores (refer to experimental participants who fall above the horizontal dotted line in Figure 2) showed more improvement on the task. A significant positive effect of *CLQT* Composite Severity was shown in accuracy for Sound to Letter Matching, Word Spelling, Sound Matching, Clock Reading, Map Reading, and Addition and in latency for Sound to Letter Matching, Word Copy, Word Spelling, and Addition. A negative effect of Composite Severity means that participants with lower than average Composite Severity scores (refer to experimental participants who fall below the horizontal dotted line in Figure 2) showed more improvement on the task. Significant negative effects were shown for accuracy in Category Matching, Feature Matching, Picture Matching, and Word Matching, and were shown for latency in Category Matching, Feature Matching, Reading Passage, Picture Matching, Word Matching, and Map Reading.

Individual participants' improvement on the therapy tasks was also examined. For this analysis, each participant's accuracy and latency reports, averaged by session, were separated out by task and level. Using this data, a separate regression analysis was completed for each level of each task each participant completed (Tables 2A–D), to determine the rate of change over time. Importantly, all participants showed significant results (either in terms of accuracy or latency) for at least one of the tasks that they were assigned during the therapy. A complete discussion of individual slopes coefficients from the regression analysis for each participant is out of the scope of this paper, however, as Table 2 shows, individual results complements the mixed model analysis discussed above.

### Between task co-improvement

All significant results from the individual regression analyses (all colored cells in Tables 2A–D) were classified by the value of the slope coefficient: for accuracy, beneficial results were considered to be any significant slope coefficient that was above zero, while for latency, beneficial results were considered to be any significant slope coefficient that was below zero. Non-significant results were not included in the co-improvement results since they cannot speak to task improvement. For all possible combinations of two tasks, the following percent of co-improvement was calculated: the number of participants with significant and beneficial slope coefficients for both tasks out of the number of participants with significant slope coefficients for both tasks. For example, examining Table 2B shows that participants 15, 18, and 21 all showed significant results from the regression analysis on both Addition and Subtraction. Participants 18 and 21 showed beneficial slope coefficients on both tasks, while participant 15 showed a beneficial slope coefficient for only one of the tasks. Therefore, the percent of co-improvement would be 66.7%. Since all participants had at least one significant result from the regression analysis, all participants were included in these calculations.

In terms of accuracy, there are three patterns of co-improvement that are apparent in Table 4A. First (refer to the top left bolded box in Table 4A), naming domain tasks such as Syllable Identification, Sound Identification and Feature Matching tend to co-improve with other naming tasks as well as with reading domain tasks such as Letter to Sound Matching, Reading Passage, and Long Reading Comprehension. Second (refer to the bottom right bolded box in Table 4A), arithmetic tasks such as Addition and Division and other quantitative reasoning tasks (Clock Math and Word Problem) tend to co-improve with other arithmetic tasks as well as with tasks in the cognitive domain such as Clock Reading, Symbol Matching, Sound Matching, Word Matching, Map Reading, Picture Ordering, Word Ordering, and Instruction Sequencing. Finally (refer to either top right or bottom left bolded boxes in Table 4A), several cognitive tasks that tap into auditory and visuospatial memory and processing but that use language stimuli such as Sound Matching, Picture Matching, and Word Matching tended to co-improve with naming and reading domain tasks such as Feature Matching, Rhyming, Syllable Identification, Reading Passage, and Word Identification. This last finding is interesting as it illustrates the intersection between language and cognitive processing. Also interesting is the observation that for some reading domain tasks such as Long Reading Comprehension and Category Identification, there is no co-improvement (refer to the top left bolded box in Table 4A).

**Table 4A:**
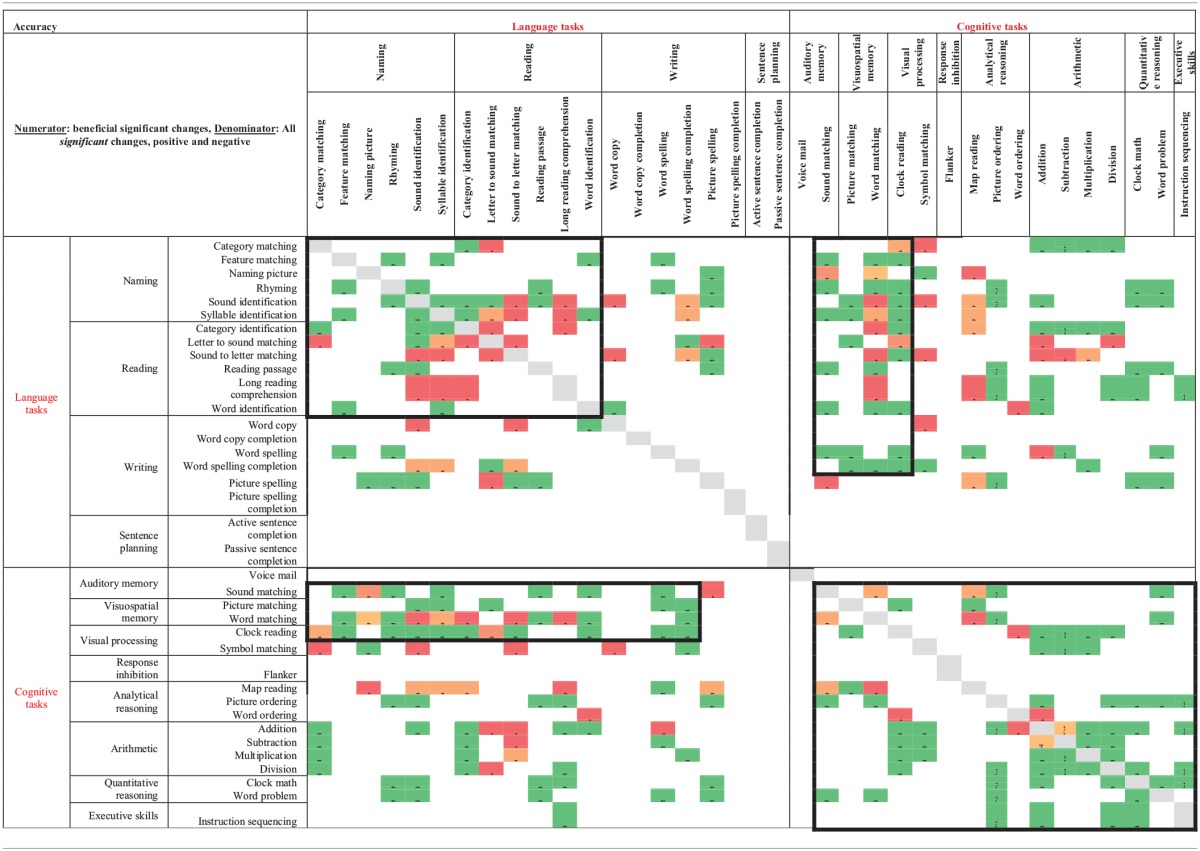
**Accuracy results from regression analysis, reporting percentage of participants that had significant slope coefficients that are beneficial out of participants that had significant slope coefficients for all combinations of two tasks (tasks organized by domain)**.

*This table is calculated based on all colored cells in Tables 2A,B. The denominator of these percentages ranges from one to four. Green denotes that 100% of all significant slope coefficients are positive; orange denotes that 25–75% of all significant slope coefficients are positive; and red denotes that 0% of all significant slope coefficients are positive. Blank cells indicate the tasks were not co-assigned for any participants or if they were co-assigned, the regression was not found to be significant*.

When examining latency (Table 4B), again three patterns of co-improvement emerged. First (refer to the top left bolded box in Table 4B), as with accuracy, changes in latency on naming tasks such as Category Matching, Feature Matching, Sound Identification, and Syllable Identification tended to co-improve with changes in latency on other naming tasks as well as with reading tasks such as Category Identification, Letter to Sound Matching, and Sound to Letter Matching. Likewise (refer to the bottom right bolded box in Table 4B), changes in latency on analytical tasks (Map Reading, Picture Ordering, and Word Ordering), arithmetic tasks (Addition, Subtraction, and Division) and a quantitative reasoning task (Word Problem) tended to co-improve. Interestingly, a third pattern emerged (refer to either top right or bottom left bolded boxes in Table 4B) where changes in latency on all cognitive domains co-improved with naming, reading and writing domains.

**Table 4B:**
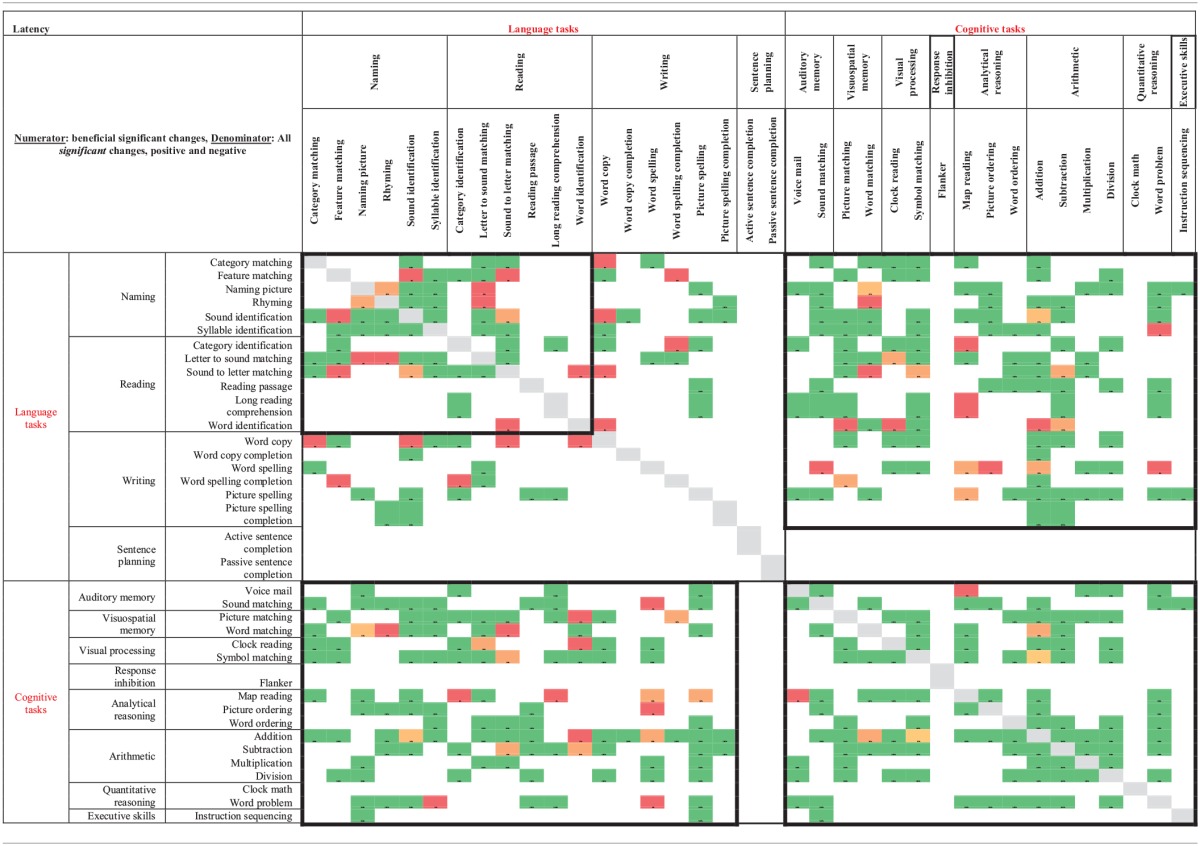
**Latency results from regression analysis, reporting percentage of participants that had significant slope coefficients that are beneficial out of participants that had significant slope coefficients for all combinations of two tasks (tasks organized by domain)**.

*This is calculated based on all colored cells in Tables 2C,D. The denominator of these percentages ranges from one to four. Green denotes that 100% of all significant slope coefficients are positive; orange denotes that 25–75% of all significant slope coefficients are positive; and red denotes that 0% of all significant slope coefficients are positive. Blank cells indicate the tasks were not co-assigned for any participants or if they were co-assigned, the regression was not found to be significant*.

### Standardized tests

Since there are unequal numbers of participants in each group, separate paired *t*-tests were done for the two groups for each of the subtests. Experimental participants showed several significant improvements from pre- to post-therapy testing. On the *R-WAB* they showed an improvement of 2.15 points on the *CQ* subtest (*t* = −2.16, *p* < 0.05) and an improvement of 3.18 points on the *AQ* subtest (*t* = −2.89, *p* < 0.01). On the *CLQT*, the experimental participants showed an improvement of 10.92 percentage points on the attention subtest (*t* = −1.93, *p* < 0.05), an improvement of 5.06 percentage points on the executive function subtest (*t* = −2.74, *p* < 0.01), an improvement of 6.89 percentage points on the visuospatial skill subtest (*t* = −3.45, *p* < 0.001), and an improvement of 5.26 percentage points on the Composite Severity score (*t* = −3.10, *p* < 0.01). The only significant change that the control participants showed was an improvement of 7.93 percentage points on the *PAPT* (*t* = −1.92, *p* < 0.05).

Correlations using pair-wise deletion of missing cases were completed to examine how individual differences (age, *MPO*, initial *R-WAB AQ* scores, and initial *CLQT* Composite Severity scores) related with changes in the standardized measures. These correlations revealed several significant relationships: intial *R-WAB AQ* scores were significantly correlated with changes in *AQ* scores (*r* = −0.33, *p* < 0.05) and with changes on the *PAPT* (*r* = −0.34, *p* < 0.05), while initial *CLQT* Composite Severity scores were significantly correlated with changes in *R-WAB CQ* scores (*r* = −0.32, *p* < 0.05), changes in the attention subtest of the *CLQT* (*r* = −0.38, *p* < 0.01), changes in the executive functions subtest of the *CLQT* (*r* = −0.31, *p* < 0.05), changes in the language subtest of the *CLQT* (*r* = −0.30, *p* < 0.05), changes in the visuospatial skills subtest of the *CLQT* (*r* = −0.34, *p* < 0.05), changes in the *CLQT* Composite Severity (*r* = −0.43, *p* < 0.01), and changes on the *PAPT* (*r* = −0.33, *p* < 0.05). These results indicated that participants with lower initial scores on the standardized measures showed more improvement on the standardized tests after therapy than participants with higher initial scores.

Additionally, independent *t*-tests were completed for all tests and subtests to test for differences between injury types for the matched pairs of participants. Significance was found for the Clock Drawing subtest of the *CLQT* (*t* = 2.83, *p* < 0.05) where the *TBI* participants show a greater change compared to the matched stroke participants. Significance was also found for the *PAPT* (*t* = −2.49, *p* < 0.05), where the matched stroke participants show a greater change compared to the *TBI* participants.

## Discussion

The goal of this study was to examine the clinical effectiveness of using an iPad-based therapy application to deliver personalized therapy to individuals with aphasia and to determine if a structured therapy program that includes homework practice, delivered through an iPad, results in significant gains in overall communication. The study also examined the effectiveness of language and cognitive therapy tasks to facilitate improvement in accuracy and latency in post-stroke and *TBI* individuals. Because the population after brain damage is invariably heterogeneous, initial scores on the *R-WAB AQ* and *CLQT* Composite Severity were chosen as two measures of language and cognitive severity to examine as covariates on outcome. In addition, age, *MPO*, injury type, and percent compliance were chosen to examine how these factors relate with therapy outcomes.

The first goal of the study was to examine if an iPad-based therapy program could be provided in a standardized but individualized manner and how therapy compliance and dosage was manifest in such a protocol. As can be seen in Tables 2A–D, it is possible to provide individualized and self-paced therapy. Notably, there was no significant difference in the therapy dosage (10 weeks of therapy for both experimental and control groups), but the amount of practice that the experimental group received was overall higher than the control group. The injury-matched pairs of participants showed no significant difference in either dosage or compliance. One interesting finding was that even though the expectation for an hour session in the clinic is that they practice the therapy for 1 h, the data from the assisted sessions demonstrates that is not the case; in fact, the actual therapy time is less than an hour (approximately 41 min), which was 68.1% compliance out of the ideal hour of clinic time. Clearly, additional opportunity to practice therapy at home increases the overall amount of therapy practice that the participant can receive and show positive behavioral changes. However, experimental participants practiced on average an additional 4 h and 8 min, rather than the ideal 6 h, ranging from zero additional home practice to 17 h and 5 min additional home practice per week. This wide range was due to the experimental design, since experimental participants, though urged to practice at home, were not forced to practice the therapy while outside of the clinic and additional practice above the ideal 6 h was not discouraged. Interestingly, participants' scores on the *R-WAB AQ* were negatively correlated with percent compliance at home, showing that participants with lower *AQ* scores had a higher compliance while at home. Future studies should experiment with different ways to encourage patients to practice the therapy more often while at home and should examine what factors may play a role in greater compliance.

The second and main goal of the current study was to examine the effectiveness of language and cognitive tasks that were provided to individual participants based on the pre-therapy behavioral profile. Both the experimental and control groups improved, validating the effectiveness of impairment-based therapy. Also, experimental participants showed more changes than control participants, especially when considering the additional homework practice that experimental participants completed. These results indicate that more therapy (i.e., an average of 4 h per week) resulted in more beneficial improvements in the rehabilitation program. While the overall number of tasks that showed beneficial changes was modest, the most effective tasks appear to be in the naming domain followed by the visuospatial memory domain. While several tasks show no significant improvement in the experimental and control group analysis (see Table 3A), it should be noted these analyses were based on the assisted sessions only, and given the nature of the mixed model analysis, the data may have been insufficient to provide a meaningful or significant result.

When looking at the scheduled and assisted sessions for just the experimental participants, several significant therapy tasks emerged for accuracy in the naming domain (3 tasks), reading domain (1 task), writing domain (1 task), and visuospatial memory (1 task). Similarly, several significant therapy tasks emerged for latency in the naming domain (2 tasks), reading domain (2 tasks), auditory memory (2 tasks), visuospatial memory (1 task). There were several tasks that showed significant non-beneficial change in terms of accuracy in the reading domain (2 tasks) and in arithmetic (1 task). A possible explanation for this may be that participants may have started therapy with higher scores on these tasks, leaving little room for improvement. There were also several tasks that showed significant non-beneficial change in latency in the reading domain (2 tasks), writing domain (1 task), arithmetic (1 task), and quantitative reasoning (1 task). A possible explanation for the latency becoming slower could be due to certain participants engaging in different strategies as a function of therapy. For instance, several participants began reading passages aloud while completing the Reading Passage task during the course of therapy, thereby slowing their latency on the task. Another possible explanation for significant non-beneficial or non significant changes in both accuracy and latency is that the analyses reported comprised tasks that are collapsed across levels. Therefore, significant effects in one level of one task may be washed out by the lack of significant effects in another level.

Because conditional improvement analyses only illustrated the overall effect of therapy without taking into account participants' individual language and cognitive profiles, the third goal was to also examine the effect of therapy after accounting for individual language (*R-WAB AQ*) and cognitive severity (*CLQT* Composite Severity) measures. From this, four sets of findings were observed. First, there are tasks that participants with low *R-WAB AQ*, or lower language skills than average (refer to all experimental participants who fall to the left of the vertical dotted line in Figure 2), benefit from the most, including Sound to Letter Matching, Word Identification, Word Spelling, Word Spelling Completion, Sound Matching, Map Reading, Addition, and Multiplication for accuracy; for latency the tasks are Letter to Sound Matching, Sound to Letter Matching, Word Copy, Word Spelling, and Multiplication. These tasks are not surprising as most of them are low level language tasks geared for participants with severe impairments (e.g., Word Identification requires the participant to match a spoken word to one of four written words). Thus, the more severely language-impaired participants tended to benefit from the simpler tasks that were assigned, which highlights the need for more impairment-based therapy. In contrast, there are tasks that participants with high *R-WAB AQ*, or higher language scores than average (refer to all experimental participants who fall to the right of the vertical dotted line in Figure 2), benefit from the most, including Letter to Sound Matching, Picture Matching, and Symbol Matching for accuracy; for latency the tasks are Syllable Identification, Category Identification, Reading Passage, Word Identification, Picture Spelling, Picture Matching, Word Matching, Map Reading, Clock Math, and Instruction Sequencing. Many of these tasks such as Picture Matching require a combination of language and short-term memory and it is not surprising that participants with better language skills did well on these tasks. Interestingly, participants with higher *R-WAB AQ* skills also showed improvement in latency on several tasks, indicating that participants with better language skills improved in their speed of response.

Next, there are tasks that participants with low *CLQT* Composite Severity, or lower cognitive skills than average (refer to all experimental participants who fall below the horizontal line in Figure 2), benefit from the most, including Category Matching, Feature Matching, Picture Matching, and Word Matching for accuracy; for latency the tasks are Category Matching, Feature Matching, Sound Identification, Syllable Identification, Reading Passage, Picture Matching, Word Matching, Clock Reading, and Map Reading. Many of these tasks require short term memory skills (including language tasks like Syllable Identification and Reading Passage), thus, changes on these tasks for participants with low cognitive skills appear reasonable. In contrast, there are tasks that participants with high *CLQT* Composite Severity, or higher cognitive skills than average (refer to all experimental participants who fall above the horizontal line in Figure 2), benefit from the most, including Sound to Letter Matching, Word Spelling, Sound Matching, Clock Reading, Map Reading, and Addition for accuracy; for latency the tasks are Sound to Letter Matching, Word Copy, Word Spelling, and Addition. Several of these tasks require memory skills (e.g., Sound Matching), although interestingly, all of these tasks rely heavily on language skills, with the exception of Clock Reading and Addition, which are less reliant on language.

Interestingly, therapy tasks where participants with high *CLQT* Composite Severity scores showed improvements were also the tasks where participants with low *R-WAB AQ* scores improved on. Specifically, this was true for Sound to Letter Matching (accuracy and latency), Word Copy (latency), Word Spelling (accuracy and latency), Sound Matching (accuracy), Map Reading (accuracy), and Addition (accuracy). The opposite effect was also seen, where participants with low Composite Severity scores and participants with high *AQ* scores both showed improvements on the same tasks including Syllable Identification (latency), Reading Passage (latency), Picture Matching (accuracy and latency), Word Matching (latency), and Map Reading (latency). Thus, these results suggest that participants with lower language skills and participants with higher cognitive skills had more to gain by way of accuracy on specific tasks whereas participants with lower cognitive skills and participants with higher language skills had more to gain by way of their speed of response. Therefore, it appears that a task like Word Spelling is well suited for participant with high cognitive skills or low language skills. Conversely, a task like Map Reading is well suited for participant with high language skills or low cognitive skills. To summarize, these results illustrate that the changes observed on the various therapy tasks can be explained by individual variability across participants and can be further examined to identify which tasks are most beneficial for participants with specific language and cognitive profiles. These results provide support to an existing literature examining the influence of severity of the language and cognitive impairment on therapy outcomes that have examined it as a *post-hoc* effect (Lee et al., 2009; Cherney, 2010; Lambon Ralph et al., 2010).

Another goal of this study was to examine tasks that tended to co-improve when assigned to the same participant over the course of the 10 week period of the study. Recall that only significantly beneficial slope effects per participant per therapy task pair were examined. Non-significant results were not included in the co-improvement results since they cannot speak to task improvement. Overall, the co-improvement results showed that reading and naming domain tended to co-improve (e.g., Letter to Sound Matching co-improved with Sound Identification in both accuracy and latency), arithmetic and quantitative reasoning tended to co-improve (e.g., Clock Math co-improved with both Addition and Division in accuracy), and several cognitive tasks in the audio and visuospatial memory domains that use linguistic stimuli co-improved with reading (e.g., Word Matching co-improved with Word Identification in both accuracy and latency) and naming (e.g., Sound Matching co-improved with Sound Identification in both accuracy and latency). While the first two observations are not surprising, they validate the theoretical premise of the therapy tasks. Indeed, previous work has shown patient-specific, task-specific co-improvement from oral reading to oral naming and vice versa (Kiran et al., 2001; Kiran, 2005). Notably, there were co-improvements between language and cognitive tasks (e.g., Picture Ordering co-improved with both Sound Identification and Syllable Identification in both accuracy and latency), which are interesting because they illustrate the intersection between language and cognitive processing skills. These results demonstrate some evidence of this intersection in gains observed after rehabilitation. Thus, far, there is compelling evidence for the intersection or interaction between language and cognitive processing (Hussey and Novick, 2012; Slevc and Novick, 2013) and preliminary evidence even in training (Kohnert, 2004). However, due to individualized and impairment-based design of the therapy (refer to Tables 2A–D), the scope of the co-improvement results is limited.

Finally, when examining changes in standardized measures due to therapy, experimental participants showed more significant and positive changes in their standardized tests than control participants. This shows that more practice on the therapy tasks resulted in greater gains in standardized measures. In fact, the only significant change in these measures that the control group showed was on the *PAPT*. During pre-therapy testing, however, the control participants had significantly different and lower scores on this test than the experimental participants and thus may have been more amenable to change as a function of therapy. The significant change on the *PAPT* is worth noting because it was expected for both groups of participants to show significant changes, but for the experimental participants to show greater change. It is, therefore, surprising that more changes were not found for the control participants.

Correlations showed that participants' initial *R-WAB AQ* scores were negatively correlated with changes in their *AQ* scores and *PAPT* scores due to therapy, meaning that participants who had lower *AQ* scores showed greater changes on *AQ* and *PAPT*. This demonstrates that participants' initial *AQ* scores were related to outcomes in measures of language impairment. Additionally, participants' initial *CLQT* Composite Severity scores were negatively correlated with changes in their *R-WAB CQ* scores, changes in their scores on several subtests of the *CLQT* (attention, executive functions, language, visuospatial skills, and Composite Severity), and changes in their *PAPT* scores due to therapy, meaning that participants who had lower Composite Severity scores showed greater changes on all of these measures. These results demonstrate that participants' initial Composite Severity scores were related to outcomes in measures of both language and cognition. These results validate the use of *R-WAB AQ* and *CLQT* Composite Severity scores as covariates in the mixed model analysis to provide information on the effect of language and cognitive skills on therapy effectiveness. The lack of correlation between changes in standardized measures and *MPO* or age, shows these factors did not play a role in how the participants changed on standardized measures due to therapy. The lack of correlation between changes in standardized measures and compliance shows that assisted compliance and scheduled compliance also did not play a role. There were, however, significant differences in changes due to therapy for two measures between the injury-matched pairs of individuals: the *TBI* participants showed greater improvement in the Clock Drawing subtest of the *CLQT* while the matched stroke participants showed greater improvement on the *PAPT*, neither of which was significantly different in pre-therapy testing. However, these results are only based on five *TBI* and matched-stroke participants, so future studies should more closely examine this difference.

Overall, both groups benefited from this therapy program (since the only group difference that was significant by domain for accuracy was in Naming and Writing). The experimental group, however, showed more improvement than the control group on standardized tests, thus, more practice with the therapy tasks resulted in greater changes. One caveat to these results is that there were many more experimental participants than control participants, and it is possible that with increasing numbers of control participants, significant differences in the standardized tests may emerge. Nonetheless, both groups of participants received similar individualized therapy programs and had approximately nine clinic one-on-one sessions over 11 weeks of therapy. The only explanation for the differences in changes on standardized tests appears to be due to the extensive practice that the experimental participants received; while control participants received an average of 40 min each week, experimental participants practiced the therapy for an average of 4 h and 49 min each week. Therefore, the increased practice time with the therapy resulted in greater and more significant gains in standardized measures, even though there was no correlation between percent compliance and changes in standardized measures.

There are several limitations to the study. As noted above, there are far fewer control participants than experimental participants. This project could not be a randomized blind study since participants were aware of whether they received the iPad extended practice or not and participants were allowed to express their willingness to participate as an experimental or control participant, especially when they owned their own iPads. This could be a source of bias because prior iPad usage may have increased effectiveness of therapy. Second, a key factor in this study was the self-paced, individualized therapy program. The strength of this study in terms of individualized therapy plans also posed methodological challenges in terms of analysis. Specifically, the co-improvement analysis is limited in the number of observations due to the individualized nature of therapy plans for each assignment. Nonetheless, preliminary trends in co-improvement provide impetus for future study of across-task changes (generalization).

There are several unanswered questions about why certain tasks appear to be effective whereas others are not. Many tasks had multiple levels of difficulty but were collapsed in the analyses reported and this could have confounded some of the results obtained. As noted above, significant effects in one level of one task may have been washed out by the lack of significant effects in another level. Future work on this data will examine task-specific, level-specific therapy outcomes. Also, because this study examined the data as mixed models, it is not possible to examine why a therapy task might not have been effective. Even though the data was examined by accounting for individual participant variability in terms of language and cognitive severity, more data is required to shed light on which specific therapy tasks work and which do not work. Since greater practice on the therapy resulted in greater gains on standardized measures, future studies should examine different ways to encourage participants to practice more often while away from the clinic when the participants are not already internally motivated to practice. Finally, examining how injury type affects responsiveness to therapy and the changes in standardized tests due to therapy should be further examined.

There are several theoretical implications of this work. While this study provided a preliminary examination of task co-improvement, the surprising pattern of task co-improvements across language and cognitive skills question the fundamental premise of training only language in individuals with post-stroke aphasia. Clearly, cognitive skills are required to perform language tasks and language skills are required to perform many cognitive tasks. Secondly, while explaining the estimate values in the mixed model analysis is out of the scope of this paper, the observation that different therapy tasks have different estimate values raises the possibility that certain tasks are generally more effective than others. Future work will examine the implication of effective and ineffective tasks from a theoretical standpoint. Finally, the software platform collected additional data on how participants used the interface buttons (such as hints or repeating instructions); however, analysis of this data was out of the scope of this paper. Future work will examine how use of the interface buttons correlate with participants' accuracy, thereby providing insights into participants' learning styles.

These results also have implications for clinical practice. For instance, Atticks describes the need for clinicians to utilize technology in a more “dynamic” way by continuing to provide support even after handing an iPad to a patient (Atticks, 2012). The ability of a clinician to utilize the application to monitor and change the therapy for participants remotely without coming to the clinic allowed the personalized self-paced progression of rehabilitation. This flexibility has implications; Van de Sandt-Koenderman suggests that with increasing use of web-based therapies, the role of the clinician will transform to becoming an “orchestrator” of their patients' rehabilitation (Van De Sandt-Koenderman, 2011). While the iPad allows patients to take some control of their therapy, as evidenced in the range of homework practiced, Holland reminds researchers that it remains important to study the effectiveness of such applications (Holland, 2014).

## Conclusion

The goal of the study was to examine the effectiveness of using an impairment-based language and cognitive therapy tasks to facilitate improvements in accuracy and latency in a group of heterogeneous post-stroke and *TBI* individuals using an iPad-based software program. In conclusion, the experimental and control groups did not differ in terms of dosage (either measured by clinic sessions or by calendar weeks in therapy), but the groups differed by compliance (time spent completing therapy tasks), with experimental participants completing more time than control participants. While both groups showed improvement, experimental participants showed more changes than control participants when examining just assisted or both types of sessions. The changes in experimental participants were possibly driven by language and cognitive standardized measures scores; notably, six tasks show effects where participants with high *CLQT* Composite Severity scores and participants with low *R-WAB AQ* scores both showed improvement. Conversely, five tasks show effects where participants with low *CLQT* Composite Severity Scores and participants with high *R-WAB AQ* scores both showed improvement. When examining how participants improve on tasks when assigned together throughout the therapy, several groupings of task co-improvement appear: reading and naming tasks, arithmetic and quantitative reasoning tasks, and language and cognitive tasks (including, memory tasks that use linguistic stimuli with reading and naming tasks). And finally, experimental participants showed more significant and positive changes in their standardized tests than control participants, showing that more practice resulted in greater and more significant gains in standardized measures and that lower initial scores on these measures were related with greater improvement on these standardized tests. These results provide preliminary but important evidence that systematic, structured, tablet-based and individualized therapy can be provided to patients.

### Conflict of interest statement

There is a significant financial relationship. Boston University owns a portion of stock equity in Constant Therapy, the software company that delivers the therapy. Carrie A. Des Roches, Isabel Balachandran and Elsa M. Ascenso own a portion of the stock equity that Boston University owns. Swathi Kiran is the co-founder and Scientific Advisor in Constant Therapy and owns stock equity in Constant Therapy. The results of the study are independent of the software platform and therefore, there is no scientific overlap. The authors declare that the research was conducted in the absence of any commercial or financial relationships that could be construed as a potential conflict of interest.

## Footnotes

^1^Assigning a task puts the task on the participants' schedule.

^2^A participants' schedule is set up and saved on the software, which can be used to start the task for both assisted and scheduled sessions.

^3^Latency reports included all items including incorrect items.

^4^Tables 2A–D contain information about the methods and individual regression results. While this does complicate the table, it was deemed more efficient than repeating the tables again with slope coefficient values from the individual regression results.

^5^Not all participants completed the 10 weeks of therapy due to schedule conflicts. These participants were not dropped from the study.

^6^When participants missed a weekly session, they would come in for a make-up session, which would often make their participation in the study last beyond 10 calendar weeks.

## Abbreviations

TBI: Traumatic brain injury
MPO: months post onset
R-WAB: Revised Western Aphasia Battery
BNT: Boston Naming Test
PAPT: Pyramids and Palm Trees
CLQT: Cognitive Linguistic Quick Test
AQ: Aphasia Quotient
CQ: Cortical Quotient.
