# Supplementary material for: Effectiveness of an impairment-based individualized rehabilitation program using an iPad-based software platform
Source: Front Hum Neurosci. 2015 Jan 5;8:1015. doi: 10.3389/fnhum.2014.01015 (PMC4283612; doi:10.3389/fnhum.2014.01015)
Supplement: Supplementary file 1 [file Table1.DOCX]

***Supplementary Material***

**Effectiveness of an impairment-based individualized rehabilitation program using an iPad-based software platform**

**Carrie Des Roches^1^*, Isabel Balachandran^1^, Elsa Ascenso^1^, Yorghos Tripodis^2^ Swathi Kiran^1^**

^1^Aphasia Research Laboratory, Sargent College, Speech, Language, and Hearing Sciences, Boston University, Boston, MA, USA

^2^Department of Biostatistics, School of Public Health, Boston University, Boston, MA, USA

*** Correspondence:** Carrie Des Roches, Aphasia Research Laboratory, Sargent College, Speech, Hearing, and Language Sciences, Boston University, 635 Commonwealth Avenue, Rm 326, Boston, MA 02215, USA.

[cadesroc@gmail.com](mailto:cadesroc@gmail.com)

1. **Supplementary Table**

**1.1 Supplementary Table 1. Table of tasks**, including: names, descriptions, number of and description of levels, cognitive/language operation, and evidence.

| **Task** | **Task Description** | **Level description** | **Cognitive/Language Operation** | **Evidence** |
| --- | --- | --- | --- | --- |
| Category Matching | Choose the category in which the presented image belongs | One level (n=685) | Semantically categorizing items to strengthen semantic representations | (Drew and Thompson, 1999),  (Kiran and Thompson, 2003), (Kiran et al., 2011) |
| Feature Matching | Yes/no questions about semantic features | Three levels with decreasing written frequency (n=1096) | Strengthening semantic representations | (Hashimoto and Frome, 2011), (Kiran et al., 2011), (Kiran and Bassetto, 2008;Kiran and Johnson, 2008), (Kiran and Thompson, 2003), (Rose and Douglas, 2008), (Stanczak et al., 2006) |
| Naming Picture | Speak the name of a presented image | Three levels with decreasing written frequency (n=685) | Retrieving semantic- phonological representations of words | (Corsten et al., 2007), (Abel et al., 2007) |
| Rhyming | Yes/no questions about whether the name of the image presented rhymes with a given word | One level (n = 590) | Retraining phonological encoding and processing | (Renvall et al., 2007), (Raymer and Ellsworth, 2002), (Doesborgh et al., 2004), (Rochon et al., 2010) |
| Sound Identification | Yes/no questions about whether the name of the image presented contains a given phoneme | One level (n=685) | Retraining phoneme processing | (Franklin et al., 2002), (Raymer and Ellsworth, 2002), (Stanczak et al., 2006), (Tessier et al., 2007), (Kendall et al., 2008) |
| Syllable Identification | Yes/no questions about whether the name of the image presented has 2 syllables | One level (n=680) | Retraining phonological segmentation | (Leonard et al., 2008), (Rochon et al., 2010) |
| Category Identification | Yes/no questions as to whether the two given words are within the same category | One level (n=683) | Distinguishing between semantically related and non-related words to strengthen semantic representations | (Kiran et al., 2009), (Sandberg and Kiran, 2014), (Raymer and Ellsworth, 2002), |
| Letter to Sound Matching | Pick the phoneme that matches the given letter | Two levels with increasing difficulty of distracters (n=52) | Retraining phoneme to grapheme conversion skills; develop sub-lexical analysis of words by identifying phonemes at the start/end of words | (Kiran et al., 2001), (Kiran, 2005), (Kiran and Viswanathan, 2008) |
| Sound to Letter Matching | Pick the letter that matches the given phoneme | Two levels with increasing difficulty of distracters (n=52) | Retraining grapheme to phoneme conversion skills; develop sublexical analysis of words by identifying phonemes at the start/end of words | (Tessier et al., 2007), (Tsapkini and Hillis, 2013), (Kiran and Viswanathan, 2008), Kiran (2005), (Kiran et al., 2001) |
| Reading Passage | Comprehend and retain information from a passage of text (recipes, weather, phone book pages, facts, menus, calendars, emails, prescriptions, online store product descriptions, and news articles) | Three levels with increased randomization of passages (n=500) | Retraining sentence and story comprehension; literacy | (Katz and Wertz, 1997), (Cherney et al., 1986) |
| Long Reading Comprehension | Comprehend and retain information from a reading passage (news articles) of roughly 225 words in order to answer a related question | Three levels with increased randomization of passages (n=247) | Retraining sentence and story comprehension; literacy | Same as Reading Passage |
| Word Identification | Pick the word heard out of multiple choices | One level (n=685) | Auditory word recognition | (Corsten et al., 2007), (Annoni et al., 1998), (Franklin et al., 2002) |
| Word Copy | Copy the written word | Five levels with increased length of words (n=685) | Retraining visuospatial skills and orthographic representation in agraphia | (Beeson et al., 2002), (Ball et al., 2011), (Beeson and Egnor, 2006), (Beeson et al., 2003), (Robson et al., 2001) |
| Word Copy Completion | Copy the written word; some letters are already filled in | Five levels with increased length of words and increased letters left blank (n=685) | Retraining visuospatial skills and orthographic representation in agraphia | Same as Word Copy |
| Word Spelling | Spell the word they hear | Five levels with increased length of words (n=685) | Retraining orthography via auditory stimuli; phonological cueing, using phoneme to grapheme conversion | (Kiran, 2005), (Rapp, 2005), (Beeson et al., 2008) |
| Word Spelling Completion | Spell the word they hear; some letters are already filled in | Five levels with increased length of words and increased letters left blank (n=685) | Retraining orthography via auditory stimuli; phonological cueing, using phoneme to grapheme conversion | Same as Word Spelling |
| Picture Spelling | Spell the word associated with the image shown | Five levels with increased length of words (n=685) | Retraining orthography via picture stimuli; phonological cueing, using phoneme to grapheme conversion | (Beeson et al., 2002), (Kiran, 2005) |
| Picture Spelling Completion | Spell the word associated with the image shown; some letters are already filled in | Five levels with increased length of words and increased letters left blank (n=685) | Retraining orthography via picture stimuli; phonological cueing, using phoneme to grapheme conversion | Same as Picture Spelling |
| Active Sentence Completion | Complete the sentence out of multiple choices | Two levels with increased difficulty of distracters (n=393) | Comprehension and production of canonical sentence structures | (Salis and Edwards, 2010) |
| Passive Sentence Completion | Complete the sentence out of multiple choices | Two levels with increased difficulty of distracters (n=393) | Comprehension and production of non-canonical sentence structures | (Stadie et al., 2008), (Weinrich et al., 2001) |
| Voice Mail | Answer the questions using an auditory voice mail | Two levels with increased randomization of auditory stimuli (n=297) | Functionally reestablishing auditory working memory skills and task related strategies | (Hart et al., 2002) |
| Sound Matching | Find all of the matching sounds | Five levels with increased number of stimuli (n=unlimited) | Incrementally retraining auditory and spatial working memory | (Westerberg et al., 2007)* training working memory |
| Picture Matching | Find all the matching pictures | Five levels with increased number of stimuli (n=unlimited) | Incrementally retraining visuospatial working memory | (Klingberg, 2010), (Westerberg et al., 2007) |
| Word Matching | Find all the matching words | Five levels with increased number of stimuli (n=unlimited) | Incrementally retraining visuospatial working memory | Same as Sound Matching |
| Clock Reading | Pick the time of an analog clock out of multiple choices | Two levels with increased difficulty of distracters (n=130) | Functionally strengthening visuospatial and spatial organization deficits via time judgment tasks |  |
| Symbol Matching | Find all the symbols that match one displayed | Ten levels with increased number of stimuli (n= unlimited) | Systematically retraining visuospatial scanning and organization skills | (Berryman et al., 2010) |
| Flanker | Choose the direction of the red arrow | One level (n=30) | Response inhibition and mental flexibility; improving selective attention | (Eriksen, 1995) |
| Map Reading | Answer multiple choice questions based on a map | Three levels with increased randomization of passages (n=468) | Multimodal interventions to reinforce visuo-perceptual, scanning, and analytical reasoning skills |  |
| Picture Ordering | Order the given pictures alphabetically based on the name of the picture | Five levels with increased number of stimuli (n=unlimited) | Multimodal intervention to improve task-related strategies while retraining analytical reasoning and working memory skills; retrieving phonological representations of words | (Vallat-Azouvi et al., 2009) |
| Word Ordering | Order the given words alphabetically | Five levels with increased number of stimuli (n=unlimited) | Multimodal intervention to improve task-related strategies while retraining analytical reasoning and working memory skills | Same as Picture Ordering |
| Arithmetic | Add/subtract/multiply/divide the given numbers | Five levels with increased number lengths (n=unlimited) | Strengthening non-linguistic cognitive processing and selective working memory deficits | (Whetstone, 1998), (Domahs et al., 2003), (Girelli et al., 1996), (Girelli and Seron, 2001) |
| Clock Math | Answer multiple choice questions about math related to time using an analog clock | Five levels with increased difficulty of operations (n=325) | Incrementally retraining quantitative reasoning skills by targeting linguistic cognitive processing, visuospatial, and working memory deficits | (Delazer et al., 1998) |
| Word Problem | Solve the given word problem | Five levels with increased number lengths, increased difficulty of distracters in questions, and increased difficulty of operations (n=unlimited) | Incrementally retraining quantitative reasoning skills by targeting linguistic cognitive processing, visual scanning, and working memory deficits | Same as Clock Math |
| Instruction Sequencing | Place written instructions (begin out of order of steps) in the correct order | One level (n=74) | Integrative reinforcement of goal directed executive functioning skills via functional planning and organization | (Ehlhardt et al., 2005) |

References

Abel, S., Willmes, K., and Huber, W. (2007). Model‐oriented naming therapy: Testing predictions of a connectionist model. *Aphasiology* 21**,** 411-447.

Annoni, J.M., Khateb, A., Custodi, M.C., Debeauvais, V., Michel, C.M., and Landis, T. (1998). Advantage of semantic language therapy in chronic aphasia: a study of three cases. *Aphasiology* 12**,** 1093-1105.

Ball, A.L., De Riesthal, M., Breeding, V.E., and Mendoza, D.E. (2011). Modified ACT and CART in severe aphasia. *Aphasiology* 25**,** 836-848.

Beeson, P.M., and Egnor, H. (2006). Combining treatment for written and spoken naming. *Journal of the International Neuropsychological Society* 12**,** 816-827.

Beeson, P.M., Hirsch, F.M., and Rewega, M.A. (2002). Successful single-word writing treatment: Experimental analyses of four cases. *Aphasiology* 16**,** 473-491.

Beeson, P.M., Rising, K., Kim, E.S., and Rapcsak, S.Z. (2008). A novel method for examining response to spelling treatment. *Aphasiology* 22**,** 707-717.

Beeson, P.M., Rising, K., and Volk, J. (2003). Writing treatment for severe aphasia: Who benefits? *Journal of Speech Language and Hearing Research* 46**,** 1038-1060.

Berryman, A., Rasavage, K., and Politzer, T. (2010). Practical clinical treatment strategies for evaluation and treatment of visual field loss and visual inattention. *NeuroRehabilitation* 27**,** 261-268.

Cherney, L.R., Merbitz, C.T., and Grip, J.C. (1986). Efficacy of Oral Reading in Aphasia Treatment Outcome. *Rehabilitation Literature* 47**,** 112-118.

Corsten, S., Mende, M., Cholewa, J., and Huber, W. (2007). Treatment of input and output phonology in aphasia: A single case study. *Aphasiology* 21**,** 587-603.

Delazer, M., Bodner, T., and Benke, T. (1998). Rehabilitation of arithmetical text problem solving. *Neuropsychological Rehabilitation* 8**,** 401-412.

Doesborgh, S.J., Van De Sandt-Koenderman, M.W., Dippel, D.W., Van Harskamp, F., Koudstaal, P.J., and Visch-Brink, E.G. (2004). Effects of semantic treatment on verbal communication and linguistic processing in aphasia after stroke: a randomized controlled trial. *Stroke; a journal of cerebral circulation* 35**,** 141-146.

Domahs, F., Bartha, L., and Delazer, M. (2003). Rehabilitation of arithmetic abilities: Different intervention strategies for multiplication. *Brain and Language* 87**,** 165-166.

Drew, R.L., and Thompson, C.K. (1999). Model-based semantic treatment for naming deficits in aphasia. *Journal of speech, language, and hearing research : JSLHR* 42**,** 972-989.

Ehlhardt, L.A., Sohlberg, M.M., Glang, A., and Albin, R. (2005). TEACH-M: A pilot study evaluating an instructional sequence for persons with impaired memory and executive functions. *Brain Injury* 19**,** 569-583.

Eriksen, C.W. (1995). The flankers task and response competition: A useful tool for investigating a variety of cognitive problems. *Visual Cognition* 2**,** 101-118.

Franklin, S., Buerk, F., and Howard, D. (2002). Generalised improvement in speech production for a subject with reproduction conduction aphasia. *Aphasiology* 16**,** 1087-1114.

Girelli, L., Delazer, M., Semenza, C., and Denes, G. (1996). The representation of arithmetical facts: Evidence from two rehabilitation studies. *Cortex* 32**,** 49-66.

Girelli, L., and Seron, X. (2001). Rehabilitation of number processing and calculation skills. *Aphasiology* 15**,** 695-712.

Hart, T., Hawkey, K., and Whyte, J. (2002). Use of a portable voice organizer to remember therapy goals in traumatic brain injury rehabilitation: A within-subjects trial. *Journal of Head Trauma Rehabilitation* 17**,** 556-570.

Hashimoto, N., and Frome, A. (2011). The use of a modified semantic features analysis approach in aphasia. *Journal of Communication Disorders* 44**,** 459-469.

Katz, R.C., and Wertz, R.T. (1997). The efficacy of computer-provided reading treatment for chronic aphasic adults. *Journal of speech, language, and hearing research : JSLHR* 40**,** 493-507.

Kendall, D.L., Rosenbek, J.C., Heilman, K.M., Conway, T., Klenberg, K., Gonzalez Rothi, L.J., and Nadeau, S.E. (2008). Phoneme-based rehabilitation of anomia in aphasia. *Brain and Language* 105**,** 1-17.

Kiran, S. (2005). Training phoneme to grapheme conversion for patients with written and oral production deficits: A model-based approach. *Aphasiology* 19**,** 53-76.

Kiran, S., and Bassetto, G. (2008). Evaluating the effectivness of semantic based treatment for naming deficits in aphasia: what works? *Seminars in Speech and Language* 29**,** 71-82.

Kiran, S., and Johnson, L. (2008). Semantic Complexity in Treatment of Naming Deficits in Aphasia: Evidence From Well-Defined Categories. *American Journal of Speech-Language Pathology* 17**,** 389-400.

Kiran, S., Sandberg, C., and Abbott, K. (2009). Treatment for lexical retrieval using abstract and concrete words in persons with aphasia: Effect of complexity. *Aphasiology* 23**,** 835-853.

Kiran, S., Sandberg, C., and Sebastian, R. (2011). Treatment of category generation and retrieval in aphasia: Effect of typicality of category items. *Journal of Speech Language and Hearing Research* 54**,** 1101-1117.

Kiran, S., and Thompson, C.K. (2003). The role of semantic complexity in treatment of naming deficits: Training semantic categories in fluent aphasia by controlling exemplar typicality (vol 46, pg 608, 2003). *Journal of Speech Language and Hearing Research* 46**,** 773-787.

Kiran, S., Thompson, C.K., and Hashimoto, N. (2001). Training grapheme to phoneme conversion in patients with oral reading and naming deficits: A model-based approach. *Aphasiology* 15**,** 855-876.

Kiran, S., and Viswanathan, M. (2008). Effect of model-based treatment on oral reading abilities in severe alexia: a case study. *Journal of Medical Speech Language Pathology.* March.

Klingberg, T. (2010). Training and plasticity of working memory. *Trends in Cognitive Sciences* 14**,** 317-324.

Leonard, C., Rochon, E., and Laird, L. (2008). Treating naming impairments in aphasia: Findings from a phonological components analysis treatment. *Aphasiology* 22**,** 923-947.

Rapp, B. (2005). The relationship between treatment outcomes and the underlying cognitive deficit: Evidence from the remediation of acquired dysgraphia. *Aphasiology* 19**,** 994 - 1008.

Raymer, A.M., and Ellsworth, T.A. (2002). Response to contrasting verb retrieval treatments: A case study. *Aphasiology* 16**,** 1031-1045.

Renvall, K., Laine, M., and Martin, N. (2007). Treatment of anomia with contextual priming: Exploration of a modified procedure with additional semantic and phonological tasks. [*http://dx.doi.org/10.1080/02687030701254248*](http://dx.doi.org/10.1080/02687030701254248) 21**,** 499-527.

Robson, J., Marshall, J., Chiat, S., and Pring, T. (2001). Enhancing communication in jargon aphasia: a small group study of writing therapy. *Int J Lang Commun Disord* 36**,** 471-488.

Rochon, E., Leonard, C., Burianova, H., Laird, L., Soros, P., Graham, S., and Grady, C. (2010). Neural changes after phonological treatment for anomia: An fMRI study. *Brain and Language* 114**,** 164-179.

Rose, M., and Douglas, J. (2008). Treating a semantic word production deficit in aphasia with verbal and gesture methods. *Aphasiology* 22**,** 20-41.

Salis, C., and Edwards, S. (2010). Treatment of written verb and written sentence production in an individual with aphasia: A clinical study. *Aphasiology* 24**,** 1051-1063.

Sandberg, C., and Kiran, S. (2014). How justice can affect jury: Training abstract words promotes generalisation to concrete words in patients with aphasia. *Neuropsychological Rehabilitation***,** 1-32.

Stadie, N., Schorder, A., Postler, J., Lorenz, A., Swoboda-Moll, M., Burchert, F., and De Bleser, R. (2008). Unambiguous generalization effects after treatment of non-cationical sentence production in German agrammatism. *Brain and Language* 104**,** 211-229.

Stanczak, L., Waters, G., and Caplan, D. (2006). Typicality-based learning and generalisation in aphasia: Two case studies of anomia treatment. *Aphasiology* 20**,** 374-383.

Tessier, C., Weill-Chounlamountry, A., Michelot, N., and Pradat-Diehl, P. (2007). Rehabilitation of word deafness due to auditory analysis disorder. *Brain injury : [BI]* 21**,** 1165-1174.

Tsapkini, K., and Hillis, A.E. (2013). Spelling intervention in post-stroke aphasia and primary progressive aphasia. *Behavioural neurology* 26**,** 55-66.

Vallat-Azouvi, C., Pradat-Diehl, P., and Azouvi, P. (2009). Rehabilitation of the central executive of working memory after severe traumatic brain injury: Two single-case studies. *Brain Injury* 23**,** 585-594.

Weinrich, M., Boser, K.I., Mccall, D., and Bishop, V. (2001). Training agrammatic subjects on passive sentences: Implications for syntactic deficit theories. *Brain and Language* 76**,** 45-61.

Westerberg, H., Jacobaeus, H., Hirvikoski, T., Clevberger, P., Ostensson, M.L., Bartfai, A., and Klingberg, T. (2007). Computerized working memory training after stroke - A pilot study. *Brain Injury* 21**,** 21-29.

Whetstone, T. (1998). The representation of arithmetic facts in memory: Results from retraining a brain-damaged patient. *Brain and Cognition* 36**,** 290-309.
